# Parallel model-based and model-free reinforcement learning for card sorting performance

**DOI:** 10.1038/s41598-020-72407-7

**Published:** 2020-09-22

**Authors:** Alexander Steinke, Florian Lange, Bruno Kopp

**Affiliations:** 1 Department of Neurology, Hannover Medical School, Carl-Neuberg-Straße 1, 30625 Hannover, Germany; 2 Behavioral Engineering Research Group, KU Leuven, Naamsestraat 69, 3000 Leuven, Belgium

**Keywords:** Human behaviour, Cognitive neuroscience, Computational neuroscience

## Abstract

The Wisconsin Card Sorting Test (WCST) is considered a gold standard for the assessment of cognitive flexibility. On the WCST, repeating a sorting category following negative feedback is typically treated as indicating reduced cognitive flexibility. Therefore such responses are referred to as ‘perseveration’ errors. Recent research suggests that the propensity for perseveration errors is modulated by response demands: They occur less frequently when their commitment repeats the previously executed response. Here, we propose parallel reinforcement-learning models of card sorting performance, which assume that card sorting performance can be conceptualized as resulting from model-free reinforcement learning at the level of responses that occurs in parallel with model-based reinforcement learning at the categorical level. We compared parallel reinforcement-learning models with purely model-based reinforcement learning, and with the state-of-the-art attentional-updating model. We analyzed data from 375 participants who completed a computerized WCST. Parallel reinforcement-learning models showed best predictive accuracies for the majority of participants. Only parallel reinforcement-learning models accounted for the modulation of perseveration propensity by response demands. In conclusion, parallel reinforcement-learning models provide a new theoretical perspective on card sorting and it offers a suitable framework for discerning individual differences in latent processes that subserve behavioral flexibility.

## Introduction

Cognitive flexibility—the ability to adjust to new task demands, rules or priorities in an adaptive manner—is considered an integral part of executive functions^1–4^. Cognitive flexibility is an important and widely studied topic in cognitive psychology. For example, there are numerous studies of cognitive flexibility in experimental psychology, often referred to as task-switching studies^5–8^. Cognitive flexibility is also of importance in studies of individual differences^9–13^. Card sorting tasks, such as the numerous variants of the Wisconsin Card Sorting Test (WCST)^14–17^, represent the gold standard for the neuropsychological assessment of cognitive flexibility^1^. Reduced cognitive flexibility on these tasks was reported in many neurological diseases^11,12,18–25^ as well as in numerous psychiatric disorders^26–29^.

**Figure 1:**
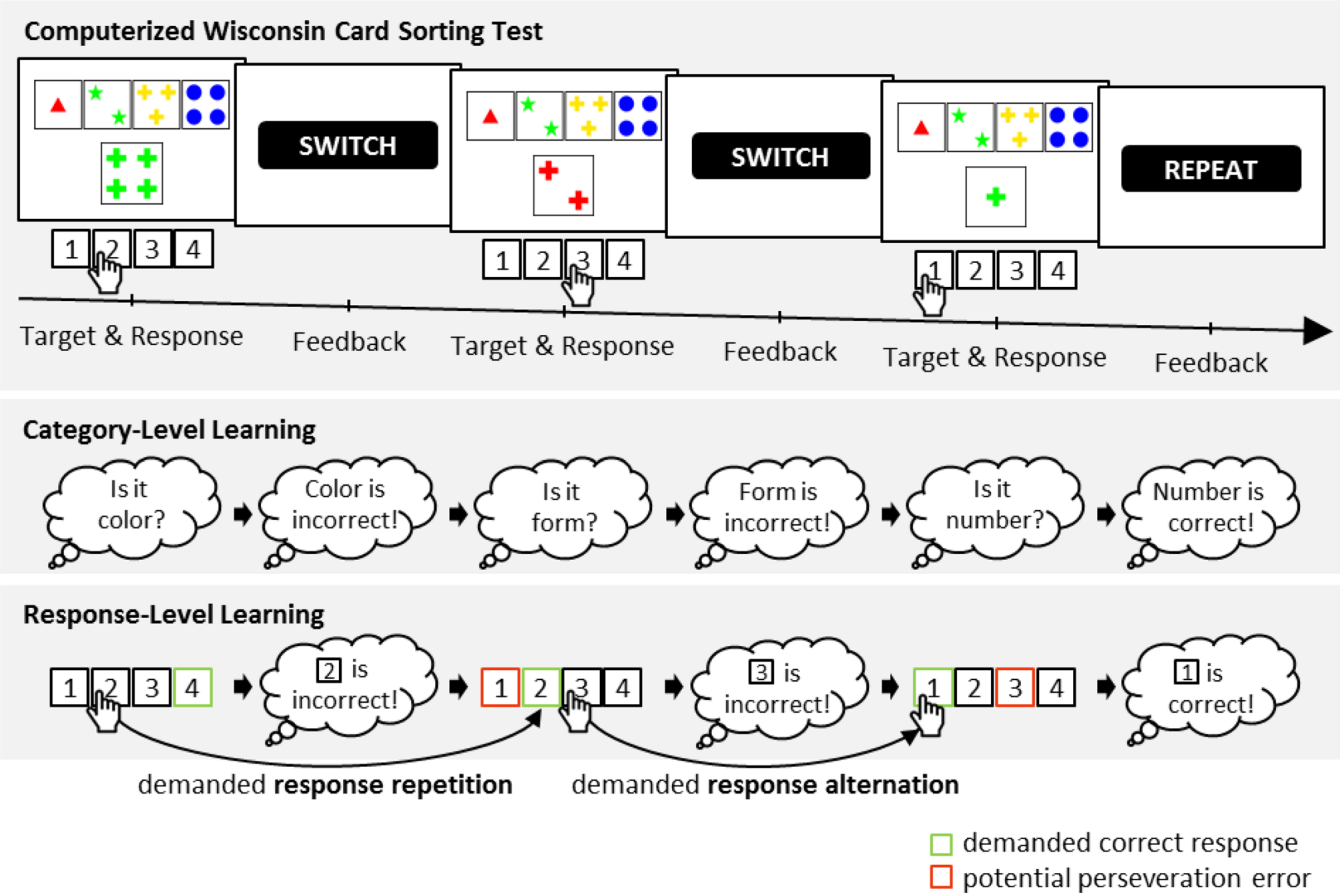
An exemplary outline of multiple levels of learning that contribute to card sorting performance. **Computerized WCST.** The present study utilizes the computerized WCST^12,35,52,53^. On the initial trial, a stimulus card (four green crosses) could be sorted according to the *color* category (inner left key card, response *2*), the *shape* category (inner right key card, response *3*), or the *number* category (far right key card, response *4*). The color category was applied, indicated by observing response *2*. A negative feedback stimulus (i.e., the visually presented word “SWITCH”) announced that this response was incorrect, meaning that the applied category should be switched. On the next trial, the stimulus card (two red crosses) was sorted by the shape category, indicated by observing response *3*. Another negative feedback stimulus announced that response *3* was incorrect, meaning that the shape category should be switched. On the next trial, the number category was applied by pressing response *1*. A positive feedback stimulus (i.e., the visually presented word “REPEAT”) indicated that response *1* was correct, meaning that the number category should be repeated on the upcoming trials. **Category-Level Learning.** Participants are supposed to consider abstract categories to guide their responses. Following negative feedback, the correct category is not yet identified and a category switch is requested. Following positive feedback, the correct category is identified and a category repetition is requested. **Response-Level Learning.** Following negative feedback, perseveration errors should be less frequent when their commitment implies repeating the previously executed responses (this can only be the case on those trial sequences that demand a response alternation; whenever a response repetition is demanded, the occurrence of an error is necessarily a response alternation). Following positive feedback, set-loss errors should be more frequent when their commitment implies repeating the previously executed responses. Kopp et al.^50^ reported asymmetrical behavioral evidence for response-level learning, namely a modulation of perseveration propensity by response demands in the absence of modulatory effects with regard to set-loss errors. Please note that we do not wish to imply that these processes are conscious (i.e., the depicted clouds might just as well reflect implicit processes).

The WCST requires participants to sort stimulus cards to key cards by categories that change periodically (see Fig. 1). In order to identify the prevailing category, participants have to rely on verbal feedback that is provided by the examiner who expresses the labels ‘correct’ (positive feedback) or ‘incorrect’ (negative feedback) on each trial. Traditional behavioral indices of card sorting performance are the number of completed categories (i.e., sequences of correct card sorts that are required to trigger a change of the correct sorting category), the number of perseveration errors (i.e., erroneous category repetitions following negative feedback), and the number of set-loss errors (i.e., erroneous category switches following positive feedback)^16,23^. Beginning with Milner’s^30^ seminal publication, perseveration errors—and to a lesser degree set-loss errors—have received by far the most attention in the field.

Manifold cognitive processes were proposed to contribute to card sorting performance, such as feedback-driven learning, category formation, set maintenance, category inference, working memory, and cognitive inhibition^1,12,31–34^. All these cognitive processes offer some degree of face validity for explaining card sorting performance. However, with the number of putative cognitive processes, and the complexity of card sorting tasks, such as the WCST, in mind, it remains difficult to infer—based on traditional methods—those cognitive processes that are truly related to card sorting performance, and how they might contribute to variability in individual card sorting performance^31^.

### Modeling individual card sorting performance

The present study relies on computational modeling. That is, it utilizes computational models to formalize hypotheses about individual cognitive processes that underlie each participant’s behavior^35–37^. Thus, one major goal of the present study is providing a computational model that offers a route towards a better understanding of individual card sorting performance. Several computational models of card sorting performance have been proposed^31,33,38–45^. Here, we focus on the computational model that yielded robust estimates of individual cognitive processes by modeling trial-by-trial responses^31,46^.

The attentional-updating (AU) model by Bishara et al.^31^ is based on a conceptualization of card sorting performance as feedback-driven learning. An attention vector represents the attentional prioritization of each category on any trial. The attention vector is updated in response to trial-by-trial feedback. The attentional category prioritizations are also related to the probability of applying a category on any trial. Individual parameters of the AU model reflect a participant’s sensitivity to positive and negative feedback, response variability (i.e., the extent to which responses reflect attentional prioritization of categories), and attentional focus (i.e., the extent to which feedback is attenuated or accentuated by attentional prioritization of categories; for a detailed account of the AU model, see section “Attentional-updating model”).

The AU model was successfully applied in clinical studies of substance dependent individuals^31^, schizophrenia^47,48^, bipolar disorder^48^, and Parkinson’s disease^35^. Individual parameter estimates were further used in a lesion mapping study that suggested an association between the presence of lesions in the right prefrontal cortex and one particular reduced model parameter, namely the sensitivity to negative feedback^49^. Simulation studies revealed that the AU model successfully recovered observed perseveration errors and set-loss errors^31,35,49^. As an interim conclusion, feedback-driven learning, as conceptualized by the AU model^31^, provides a computational model of individual card sorting performance that is consistent with a number of behavioral findings.

### Re-conceptualizating card sorting performance

Behavioral findings from a recent study^50^ suggest that multiple levels of learning contribute to card sorting performance (see Fig. 1; note that Fig. 1 considers the computerized WCST (cWCST), which was utilized in the present study). It is commonly assumed that trial-by-trial feedback triggers category-level learning: Category-level learning implies that participants switch between suitable categories on trials following negative feedback, and that they maintain categories on trials following positive feedback. The occurrence of perseveration errors and set-loss errors are commonly considered as behavioral indices of unsuccessful category-level learning. Our analysis of card sorting performance is novel in so far as it considers that trial-by-trial feedback might also trigger response-level learning. Response-level learning implies that participants tend to avoid the previously executed response following negative feedback. They may also tend to repeat the previously executed response following positive feedback.

Behavioral evidence for the existence of response-level learning was reported in our previous study^51^. In particular, perseveration errors occurred less frequently when their occurrence implied repeating the previously executed response (see the “demanded response alternation” trial sequence depicted in Fig. 1; here, perseveration errors occur on response repetition trials) compared to when their occurrence did not imply repeating the previous response (see the “demanded response repetition” trial sequence depicted in Fig. 1; here, perseveration errors occur on response alternation trials). Hence, the propensity of committing a perseveration error was modulated by response demands: The occurrence of perseveration errors became less likely when it implied repeating the response that had received an incorrect feedback on the previous trial. In contrast, no evidence for a modulation of set-loss propensity by response demands was found: Set-loss errors did not occur more frequently when they implied repeating the response that received a correct feedback on the previous trial. Thus, the novel finding of a modulation of the perseveration propensity by response demands could provide a behavioral indicator of response-level learning on the WCST.

### A reinforcement-learning model of individual card sorting performance

To integrate the novel behavioral evidence into a computational model of card sorting performance, we utilize the well-known mathematical framework of reinforcement learning^54^. Reinforcement learning describes how actions (e.g., responses on the cWCST) are selected in the face of positive and negative feedback^54–60^. Reinforcement learning is based on the assumption that participants form feedback expectations of actions, and that stronger expectations of positive feedback are associated with a higher probability of executing the corresponding action. Importantly, feedback expectations of executed actions are updated in response to feedback, with the strength of updating being modulated by prediction errors that equal the difference between the obtained feedback and expected feedback: Large prediction errors are associated with stronger updating of feedback expectations. Typical individual parameters are learning rates after positive and negative feedback (i.e., the extent to which prediction errors are integrated into feedback expectations), and a temperature parameter (i.e., the extent to which executed actions accord to current feedback expectations). Here, we propose for the first time that reinforcement learning provides a suitable computational framework for modeling card sorting performance.

Dual-level models present a prominent approach of modeling multiple levels of reinforcement learning^61–66^. Model-based (MB) reinforcement learning operates at an abstract level, which guides selection of task-appropriate actions, while model-free (MF) reinforcement learning bypasses the abstract level. Here, actions that were followed by positive feedback tend to be repeated, whereas actions that were followed by negative feedback tend to be avoided. We introduce parallel reinforcement-learning models of card sorting performance that incorporate parallel MB- and MF-reinforcement learning in an attempt to account for individual card sorting performance, including the newly discovered modulation of perseveration propensity by response demands.

### Primary study aims

One aim of the current study is replicating the previously reported modulation of perseveration propensity by response demands. Kopp et al.^50^ analyzed data from a sample of brain-damaged inpatients (*N* = 112) using the Modified-WCST (M-WCST)^67^. The M-WCST is a short paper-and-pencil variant of the WCST (comprising a maximum of six switches of the correct sorting category). Participants are required to physically sort stimulus cards to key cards, followed by verbal feedback (“correct” vs. “incorrect”) that was provided by the examiner. It remains an open question whether the reported modulation of perseveration propensity by response demands generalizes to card sorting performance on other WCST versions. Therefore, we tested whether the reported modulation of perseveration propensity by response demands is replicable on the cWCST. In addition, the possibility cannot be excluded that the modulation of perseveration propensity by response demands may be exclusively observable in brain-damaged patients. In this study, we aim to address these questions by analyzing data from a large sample of non-clinical participants (*N* = 375 undergraduates) who completed a computerized variant of the WCST^52^. The cWCST that was utilized in the present study^12,35,53,68^ (see Fig. 1) includes as many as 41 switches of the correct sorting category (rather than up to six switches of the correct category in the M-WCST). On the cWCST, participants respond via key presses, followed by visual feedback cues (“switch” vs. “repeat” rather than “incorrect” and “correct”).

The major aim of the current study is providing a suitable cognitive theory of card sorting performance by means of novel parallel reinforcement-learning models. Cognitive theories of card sorting performance should be able to account for a wide range of behavioral effects that are detectable on card sorting tasks. Hence, the benchmark for all computational models under consideration is the successful recovery of perseveration and set-loss error propensities as well as the novel modulation of perseveration errors by response demands. In order to test whether parallel reinforcement-learning models represent better computational models than a single-level reinforcement-learning model, we compare their performance with that of a pure model-based reinforcement-learning (MB-RL) model. In addition, we compare the performance of these reinforcement-learning models with the performance of the state-of-the-art AU model^31^. Model performance was firstly assessed by estimating predictive accuracies. However, analyzing predictive accuracy is not informative with regard to whether a computational model recovers the behavioral phenomena of interest^69^. Therefore, we also simulated individual participants’ behavior using each of the three computational models and its individual parameter estimates.

## Results

### Behavioral analysis

For analysis of behavioral card sorting data, traditional set-loss errors (a switch of the applied category after positive feedback) and perseveration errors (a repetition of the applied category after negative feedback) served as outcome measures. We considered set-loss and perseveration errors as behavioral indicators of the efficacy to adapt card-sorting behavior to negative and positive feedback cues (i.e., to switch the applied category after negative feedback and to repeat the applied category after positive feedback, respectively). Thus, we considered perseveration and set-loss errors appropriate for evaluations of the novel reinforcement-learning models, which are based on a conceptualization of card sorting performance as feedback-driven learning. As Kopp et al.^50^ did with a traditional paper-and-pencil version of the WCST^67^, we stratified these error scores by response demands (i.e. repetition vs. alternation; see Fig. 1). A demanded response repetition was scored if the correct response (i.e., responses that repeated a category after positive feedback or responses that switch the category after negative feedback) matched the executed response on trial *t *− 1. A demanded response alternation was scored when the incorrect response (i.e., responses that switch the category after positive feedback or responses that repeat the category after negative feedback) matched the executed response on trial *t *− 1 (see Fig. 1). Conditional error probabilities were computed by dividing the number of committed errors by the number of trials on which the respective error type was possible. Conditional error probabilities were entered into a Bayesian repeated measures analysis of variance (ANOVA) with the factors error type (set-loss vs. perseveration) and response demand (repetition vs. alternation).

Results of the Bayesian repeated measures ANOVA are presented in Table 1. The ANOVA model including both main effects and the interaction effect of error type and response demand was most likely given the data. Inspection of Fig. 3 (upper left plot) revealed a generally higher perseveration propensity than set-loss propensity. Conditional perseveration error probabilities were reduced with a demanded response alternation when compared to a demanded response repetition. This finding replicates the M-WCST-based finding of a modulation of perseveration propensity by response demands^50^.

**Table 1:** Results of Bayesian repeated measures ANOVAs for observed and simulated conditional error probabilities.

ANOVA model	Observed		Simulated							
			AU		MB-RL		P-RL		wP-RL	
	P(M|D)	log(BF_M_)	P(M|D)	log(BF_M_)	P(M|D)	log(BF_M_)	P(M|D)	log(BF_M_)	P(M|D)	log(BF_M_)
ET + RD + ET*RD	**> 0.999**	**33.26**	0.005	− 3.89	0.004	− 4.04	**> 0.999**	**16.45**	** > 0.999**	**15.38**
ET + RD	< .001	− 30.48	0.057	− 1.42	0.059	− 1.37	< 0.001	− 13.80	< 0.001	− 12.75
ET	< 0.001	− 40.79	**0.938**	**4.11**	**0.936**	**4.07**	< 0.001	− 17.22	< 0.001	− 14.68
RD	< 0.001	− 89.44	< 0.001	− 101.06	< 0.001	− 134.08	< 0.001	− 107.04	< 0.001	− 79.70
Null model	< 0.001	− 98.38	< 0.001	− 98.26	< 0.001	− 130.78	< 0.001	− 108.36	< 0.001	− 81.05

Most likely ANOVA model given the data in bold.

*AU *attentional-updating model, *MB-RL *only model-based reinforcement-learning model, *P-RL *parallel reinforcement-learning model, *wP-RL *weighted parallel reinforcement-learning model, *P*(*M|D*) posterior probability of ANOVA model (M) given the data (D), *log*(*BF*_*M*_) logarithmized Bayes factors for any ANOVA model when compared to all the other ANOVA models together, *ET *factor error type (set-loss vs. perseveration), *RD *factor response demand (repetitions vs. alternation), *null model *ANOVA model including neither main effects nor the two-way interaction.

### Computational modeling

The parallel reinforcement-learning models incorporate MB- and MF-reinforcement learning. MB-reinforcement learning operates on feedback expectations for the application of categories, which are updated in response to feedback and subsequently used to guide responses. In contrast, MF-reinforcement learning directly operates on feedback expectations of responses irrespective of corresponding sorting categories. For any trial, feedback expectations of MB- and MF-reinforcement learning are linear integrated and response probabilities are derived from these integrated feedback expectations. Individual parameters of the parallel reinforcement-learning models are MB- and MF-learning rates. In order to account for different strengths of learning from positive and negative feedback, MB- and MF-learning rates are further separated for trials following positive and negative feedback. The parallel reinforcement-learning models also incorporate individual MB- and MF-inertia parameters, which quantify the impact of previous feedback expectations on current responding^70,71^. Lastly, an individual temperature parameter gives the extent to which responding accords to integrated feedback expectations.

We considered two configurations of parallel reinforcement-learning models. First, the wP-RL (weighted parallel reinforcement-learning) model incorporates an individual weighting parameter^61,72^, which quantifies the relative strength of MB- over MF-reinforcement learning. Second, we considered a less complex configuration of the wP-RL model, i.e., the P-RL model. In the P-RL model, feedback expectations of MB- and MF-reinforcement learning are linear integrated without any weighting. Instead, MB- and MF-reinforcement learning might be indirectly weighted by means of relative heights of learning rate parameters (i.e., generally higher MB-learning rates than MF-learning rates cause MB-feedback expectations to be higher than MF-feedback expectations, and vice versa).

In summary, we considered four computational models of card sorting performance, i.e., the wP-RL model, the P-RL model, the MB-RL model including only MB-reinforcement-learning, and the state-of-the-art AU model^31^. Analyses of parameter correlations, parameter recovery and model recovery are presented in the Supplementary Materials.

#### Relative model performance

We assessed a computational model’s performance by Bayesian *K*-fold cross validation as an indicator of a model’s predictive accuracy. Bayesian *K*-fold cross validation quantifies a model’s predictive accuracy by the estimated log predictive density (elpd). Following, relative model performance was quantified by the difference in elpd between the model with the lowest absolute elpd and any other model (Δelpd). The lower the absolute elpd, the better is a model’s performance (i.e., a better predictive accuracy). Hence, larger absolute Δelpd-values indicate worse model performance (for details, see “Methods”, “Relative model performance”).

Group-level relative model performance results are presented in Table 2. The wP-RL model showed the best predictive accuracy (elpd = − 37,412) followed by the P-RL (Δelpd between the wP-RL and the P-RL model = − 161; *SE* = 23) and the MB-RL model (Δelpd between the wP-RL and the MB-RL model = -301; *SE* = 46). All reinforcement-learning models (i.e., the wP-RL, the P-RL, and the MB-RL model) outperformed the state-of-the-art AU model^31^, which should be considered as the benchmark for model comparison (Δelpd between the wP-RL and the AU model = − 2,857; *SE* = 118; Δelpd between the P-RL and the AU model = − 2,696; *SE* = 112; Δelpd between the MB-RL and the AU model = − 2,556; *SE* = 118).

**Table 2:** Group-level results of Bayesian *K*-fold cross validation.

Computational model	Parameter	elpd		Δelpd	
wP-RL	8	− 37,412	(782)		
P-RL	7	− 37,573	(785)	− 161	(23)
MB-RL	4	− 37,713	(797)	− 301	(46)
AU	4	− 40,269	(805)	− 2,857	(118)

*Parameter *number of free parameters, *elpd *estimated log predictive density, *Δelpd *difference in estimated log pointwise predictive density between a model and the best performing model; standard error in parentheses, *AU *attentional-updating model, *MB-RL *only model-based reinforcement-learning model, *P-RL *parallel reinforcement-learning model, *wP-RL *weighted parallel reinforcement-learning model.

**Figure 2:**
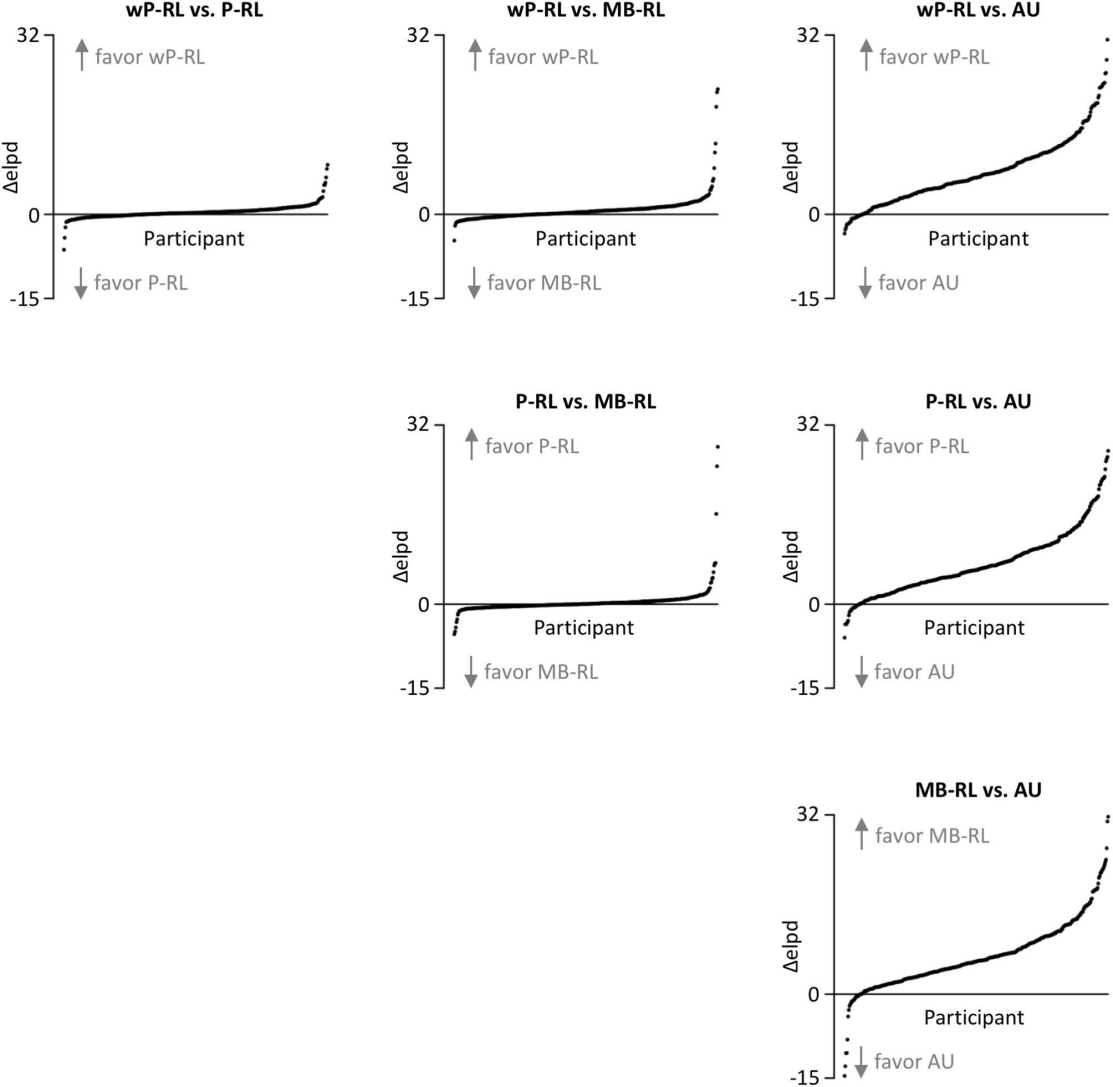
Individual-level results of *K*-fold cross validation. Dots represent single participants. *Δelpd *individual difference in estimated log pointwise predictive density between models under consideration, *AU *attentional-updating model, *MB-RL *only model-based reinforcement-learning model, *P-RL *parallel reinforcement-learning model, *wP-RL *weighted parallel reinforcement-learning model.

Individual-level relative model performance results are depicted in Fig. 2. In general, the wP-RL model performed best for 56% of all participants and the P-RL model for 15% of all participants. In contrast, the MB-RL and the AU model performed best for 26% and 3% of all participants, respectively. Pairwise model comparisons, which are depicted in Fig. 2, revealed that the wP-RL, the P-RL, and the MB-RL model showed better predictive accuracies than the AU model for 93%, 94%, and 94% of all participants, respectively. Thus, all reinforcement-learning models outperformed the state-of-the-art AU model on an individual-level. With regard to comparisons of individual predictive accuracies between reinforcement-learning models, the wP-RL model performed better than the P-RL model for 69% of all participants. The wP-RL model performed also better than the MB-RL model for 69% off all participants. Hence, the wP-RL model provided better predictive accuracies than other reinforcement-learning models for most participants. The P-RL model performed better than the MB-RL model for 53% of all participants.

#### Absolute model performance

Relative model comparisons are not informative about a model’s ability to simulate the behavioral phenomena of interest^69^. Therefore, we assessed absolute model performance by simulating individual card sorting behavior according to the post-hoc absolute fit method^73^, which is appropriate for analyses of reinforcement-learning models (see Konstantinidis et al.^74^ for a detailed discussion). Simulated card sorting behavior was analyzed by means of conditional error probabilites.

**Figure 3:**
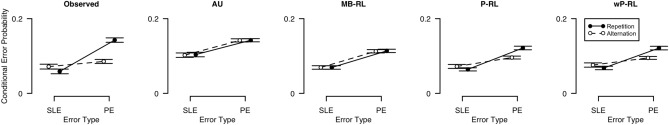
Observed (left plot) and simulated group mean conditional error probabilities (all other plots). Error bars indicate ± 1 standard error of the mean. Note that set-loss and perseveration errors follow positive and negative feedback, respectively. *AU *attentional-updating model, *MB-RL *only model-based reinforcement-learning model, *P-RL *parallel reinforcement-learning model, *wP-RL *weighted parallel reinforcement-learning model, *SLE *set-loss error, *PE *perseveration error, *Repetition *demanded response repetition, *Alternation *demanded response alternation, *Conditional error probability *probability of an error given error type (perseveration vs. set-loss) and response demand (repetition vs. alternation).

Results of Bayesian repeated measures ANOVAs (see Table 1) revealed that only data simulated by the wP-RL and the P-RL model mirrored the results of the analysis of observed data, i.e., the most likely ANOVA model given the data included both main effects and the two-way interaction of error type and response demand. For the MB-RL model and the AU model, the most likely ANOVA model given the data included only the main effect of error type. Inspection of Fig. 3 reveals that all considered computational models were able to simulate the finding of generally higher perseveration propensity than set-loss propensity. Importantly, only the wP-RL and the P-RL model simulated the modulation of perseveration propensity by response demands. Thus, combining MB- and MF-reinforcement learning as in the wP-RL and the P-RL model appears to successfully account for the modulation of perseveration propensities by response demands.

**Figure 4:**
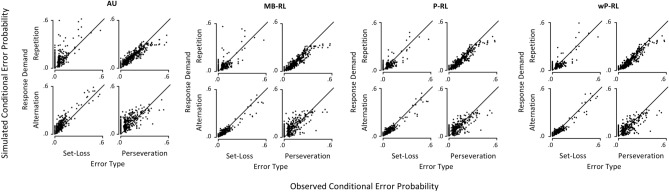
Observed (*x*-axis) and simulated (*y*-axis) individual conditional error probabilities. Note that set-loss and perseveration errors follow positive and negative feedback, respectively. *AU *attentional-updating model, *MB-RL *only model-based reinforcement-learning model, *P-RL *parallel reinforcement-learning model, *wP-RL *weighted parallel reinforcement-learning model, *Conditional error probability *probability of an error given error type (perseveration vs. set-loss) and response demand (repetition vs. alternation).

Group-level analyses of simulated behavioral performance indices are not informative about whether a computational model presents a good description of individual behavioral performance indices. Thus, we depicted the recovery of individual conditional error probabilities in Fig. 4. In order to quantify a computational model’s ability to account for inter-individual variance of behavioral performance indices, we computed *R*^2^ statistics of observed conditional error probabilities when predicted by simulated conditional error probabilities using Bayesian linear regression analysis.

Results are presented in Table 3. For all behavioral performance indices under consideration, the wP-RL model presented a similar or even higher *R*^2^ statistics than the P-RL model. The MB-RL model showed lower *R*^2^ statistics than both the wP-RL and the P-RL model. The AU model showed the lowest *R*^2^ statistics. Thus, with regard to all computational models that were under consideration, the wP-RL and the P-RL model recovered the highest amount of inter-individual variance of behavioral performance indices.

**Table 3:** Recovery of inter-individual variance of behavioral indices given by the *R*^2^ statistic.

Error type	Response demand	Computational model			
		AU	MB-RL	P-RL	wP-RL
Set-loss error	Repetition	0.61	0.68	0.79	0.80
	Alternation	0.83	0.92	0.94	0.94
Perseveration error	Repetition	0.80	0.84	0.86	0.86
	Alternation	0.48	0.53	0.59	0.60

*AU *attentional-updating model, *MB-RL *only model-based reinforcement-learning model, *P-RL *parallel reinforcement learning model, *wP-RL *weighted parallel reinforcement-learning model.

#### Parameter estimation

The wP-RL model performed best by means of relative and absolute model performance. Group-level parameter estimates of the wP-RL model are presented in Table 4. Estimates of the weighting parameter indicated a stronger weighting of MF-reinforcement learning when compared to MB-reinforcement learning. However, learning rates of MB-reinforcement learning were higher than learning rates of MF-reinforcement learning. For MB-reinforcement learning, the learning rate after positive feedback was higher than the learning rate after negative feedback, whereas the opposite occurred for MF-reinforcement learning. In fact, the learning rate of MF-reinforcement learning after positive feedback was close to zero, indicating that MF-feedback expectations of responses were virtually not updated after positive feedback. Inertia parameters were roughly equal for MB- and MF-reinforcement learning and substantially different from 1, indicating that inertia of feedback expectations contributed to model performance (i.e., a parameter value of 1 would indicate that no inertia happens from trial-to-trial). The temperature parameter was smaller than 1, indicating that differences in integrated feedback expectations were accentuated for computing response probabilities.

**Table 4:** Summary statistics of group-level parameter estimates of the wP-RL model.

Parameter	Description	Mean	*SD*	95% HDI	
				Lower	Upper
$\alpha_{MB}^{ + }$	Model-based learning rate after positive feedback	> 0.99	< 0.01	0.99	> 0.99
$\alpha_{MB}^{ - }$	Model-based learning rate after negative feedback	0.60	0.03	0.55	0.66
$\gamma_{MB}$	Model-based inertia	0.31	0.02	0.28	0.34
$\alpha_{MF}^{ + }$	Model-free learning rate after positive feedback	< 0.01	< 0.01	< 0.01	< 0.01
$\alpha_{MF}^{ - }$	Model-free learning rate after negative feedback	0.02	0.01	0.01	0.03
$\gamma_{MF}$	Model-free inertia	0.35	0.06	0.22	0.46
$\tau$	Temperature	0.09	0.02	0.06	0.11
*w*	Weighting of model-based and model-free RL	0.33	0.06	0.20	0.42

Posterior distributions of Probit-transformed group-level location parameters from hierarchical Bayesian analysis are reported.

*RL *reinforcement learning, *SD* standard deviation, *95% HDI *95% highest density interval.

## Discussion

The results from the present study of card sorting replicate the previously reported modulation of perseveration propensity by response demands, in the absence of a modulation of set-loss errors by response demands^50^. Perseveration errors were less likely when their occurrence implied repeating the response that had received an incorrect feedback on the previous trial. These findings were obtained from a relatively large sample of young participants. The results suggest that the original findings are neither specific for brain-damaged patients nor specific for particular card sorting tasks such as the M-WCST. We introduced parallel reinforcement-learning models that incorporate MB- and MF-reinforcement learning to account for individual card sorting performance. Our results indicate that parallel reinforcement-learning models outperform competing computational models of card sorting performance. Only parallel reinforcement-learning models recovered the modulation of perseveration propensity by response demands, which demonstrates the advantage of combining MB- and MF-reinforcement learning over purely MB-reinforcement learning (i.e., the MB-RL model). Furthermore, all reinforcement-learning models under consideration outperformed the AU model: Reinforcement learning seems to provide a generally more suitable framework for understanding card sorting performance than does the state-of-the-art AU model^31^.

We replicated the modulation of perseveration propensity by response demands in a large sample of young participants using the cWCST. The successful replication of that behavioral phenomenon should be interpreted with regard to differences between the M-WCST, which was utilized in the previous study^50^, and the cWCST, which was utilized in the present study. First, the M-WCST consists of 48 trials (including up to six switches of the correct category), resulting in a relatively low number of occasions on which an error type of major interest (i.e., perseveration errors) may occur. Administering the cWCST raises these numbers because it consists of a fixed number of 41 switches of the correct category, yielding a relatively large number of trials overall (*M* = 168 trials; *SD* = 14 trials; note that the individual number of trials, which is needed to complete 41 switches of the correct category, depends on the overall performance of a participant). Second, there are a number of qualitative differences between the M-WCST and the cWCST. These qualitative differences are: Type of feedback (verbally uttered “correct”- or “incorrect”-feedback vs. visually presented “repeat”- or “switch”-feedback), duration of key card presentation (constantly present key cards vs. only present on the screen while stimulus cards are presented), type of responses (direct spatial match between stimulus card and key card vs. pressing spatially allocated buttons), and the general setting (manual administration vs. computerized administration). Previous research remained inconclusive as to what extent behavioral performance indices obtained from manual and computerized WCST versions are comparable^75–77^. Replicating the modulation of perseveration propensity by response demands implies that this novel behavioral phenomenon seems to be generalizable across manifold versions of card sorting tasks. The successful replication of the modulation of perseveration propensity by response demands in a large sample of young participants also suggests that this behavioral phenomenon in card sorting data is robustly detectable from individuals with no known brain damage.

The successful replication of the modulation of perseveration propensities by response demands sheds new light on the interpretation of perseveration errors. Perseveration errors are traditionally interpreted as indices of cognitive inflexibility, i.e., failures to shift away from abstract sorting categories^12^. Our results suggest that perseveration errors may not be considered as ‘pure’ indices of cognitive inflexibility. Instead, as perseveration error propensities seem to be modulated by response demands, it appears that another learning process contributes to the occurrence of perseveration errors as well. This learning process might be best described as response-level learning.

We propose a suitable cognitive theory of card sorting performance by means of novel parallel reinforcement-learning models. Parallel reinforcement-learning models integrate reinforcement learning that occurs in parallel at the MB-category level and at the MF-response level. The need for combining MB- and MF-reinforcement learning was suggested by our model comparisons, in which the wP-RL and the P-RL model outperformed pure MB-reinforcement learning (i.e., the MB-RL model) in terms of absolute (i.e., simulation of participants’ behavior) model performance. Only the wP-RL and the P-RL model recovered all the behavioral phenomena under consideration, i.e., the generally higher perseveration propensity than set-loss propensity, and the modulation of perseveration propensity by response demands. Thus, combining MB- and MF-reinforcement learning appears to have an edge over pure MB-reinforcement learning with regard to the successful recovery of the modulation of perseveration propensity by response demands. In contrast, as the pure MB-reinforcement learning model as well as the AU model failed to recover the modulation of perseveration propensity by response demands, these computational models should be considered insufficient cognitive theories of card sorting performance.

The wP-RL and the P-RL model outperformed both the MB-RL and the AU model. However, the wP-RL and the P-RL model could have performed best simply due to their relatively high complexity, i.e., they incorporate more individual model parameters than their competitors (eight or seven free parameters, respectively, vs. four free parameters in both the MB-RL and the AU models). The question emerges as to how the more complex wP-RL and P-RL models can be justified compared to the more parsimonious models of card sorting performance, i.e., the MB-RL and the AU model. We assessed model performance by means of predictive accuracies. Model parameters that merely fit non-replicable, idiosyncratic noise in the data exert detrimental effects on the model’s predictive accuracy (for a detailed discussion, see “Methods”, “Relative model performance”). On the group-level, the predictive accuracy of the wP-RL model (and of the P-RL model) was superior to the predictive accuracies of the MB-RL and the AU model. However, on an individual-level, the parallel reinforcement-learning models performed best for only 71% of all participants (56% and 15% for the wP-RL and the P-RL model, respectively). Thus, predictive accuracies indicate that parallel reinforcement-learning models performed best for most participants but not for all participants. Importantly, only the parallel reinforcement-learning models recovered the modulation of perseveration propensity by response demands. Thus, the additional model parameters of the parallel reinforcement-learning models, which were mainly introduced by the MF-reinforcement-learning algorithm, seem to be necessary to account for this well-replicable behavioral phenomenon. Future research should address how the complexity of parallel reinforcement-learning models could be reduced without worsening their predictive accuracies, while maintaining their ability to recover all studied behavioral phenomena of card sorting. Possible ways to reduce the number of parameters of the parallel reinforcement-learning models are outlined throughout the remainder of the “Discussion”.

As mentioned in the Introduction, many neurological diseases and psychiatric disorders are associated with elevated perseveration propensities on the WCST. However, heightened numbers of perseveration errors in card sorting tasks are just a non-specific behavioral symptom of these conditions^12^. The low specificity of behavioral card sorting symptoms (such as elevated perseveration propensity) should be resolved with regard to separable cognitive processes^1,12,31,78^. Pursuing a computational approach might provide methods for disentangling those processes that could be specifically affected by neurological diseases and psychiatric disorders. For example, elevated perseveration propensity that occurs in patients who suffer from a particular diagnosis might be associated with increased inertia of MB-feedback expectations (i.e., heightened $\gamma_{MB}$), or with lowered MB learning from negative feedback (i.e., lowered $\alpha_{MB}^{ - }$). In contrast, elevated set-loss propensity might be associated with decreased inertia of MB-feedback expectations (i.e., lowered $\gamma_{MB}$), or with lowered MB learning from positive feedback (i.e., lowered $\alpha_{MB}^{ + }$). In contrast, the overall error propensity might be increased due to generally heightened contribution of MF learning (i.e., increased $\alpha_{MF}^{ + }$ and $\alpha_{MF}^{ - }$), or due to less consistent responding (i.e., increased $\tau$). Strong modulations of perseveration propensity by response demands might be specifically associated with increased inertia of MF-feedback expectations (i.e., increased $\gamma_{MF}$), or with heightened MF-learning rate after negative feedback (i.e., increased $\alpha_{MF}^{ - }$). These examples illustrate that future computational research of card sorting might contribute a better understanding of behavioral card sorting symptoms (for an illustrative example of the effect of model parameters on feedback expectations, see^79,80^).

Comparisons of relative model performance indicated that the wP-RL model provided the best group-level predictive accuracy, followed by the P-RL and the MB-RL model. On the individual-level, the parallel reinforcement-learning models performed best for 71% of all participants (i.e., 56% and 15% for the wP-RL and the P-RL models, respectively). For 56% of all participants, the wP-RL model showed best predictive accuracies. However, results of model recovery analysis (see Table S9) indicate that the wP-RL model can show the best predictive accuracy for card sorting performance that was actually generated by the P-RL model. Thus, it remains possible that card sorting performance of these participants was actually better conceptualized by the P-RL model than by the wP-RL model. For 26% of all participants, the solely MB-reinforcement-learning model performed best. These results suggest that parallel reinforcement-learning models might not provide the best description of card sorting performance for all participants. In contrast, cWCST performance of a subset of participants was better described by a solely MB-reinforcement-learning model. Thus, it remains possible that not all participants show category- and response-level learning on the cWCST (as indicated by a best-fitting MB-RL model). Instead, a non-negligible subset of participants might show virtually no response-level learning on the cWCST.

The wP-RL model provided the better group-level predictive accuracy when compared to the P-RL model. On the individual level, the wP-RL model performed better than the P-RL model for 69% of all participants. Thus, analyses of predictive accuracies suggest that the wP-RL model, which includes an additional weighting parameter that arbitrates between MB- and MF reinforcement-learning, outperforms the P-RL model. However, the wP-RL model did not sufficiently recover parameters from simulated data (see Figure S1). Thus, the studied cWCST might be underpowered to assess the wP-RL model appropriately^81^. In contrast, the P-RL model recovered parameters from simulated data reliably (see Figure S2). Hence, future studies of individual differences in parameters of parallel reinforcement-learning models should utilize the P-RL model.

How feedback cues on the cWCST should be understood in a reinforcement-learning framework needs some conceptual clarification. Reinforcement-learning frameworks describe action selection in the face of reward or punishment. In experimental studies of reinforcement learning, reward (or punishment) is typically implemented as monetary incentives^82^ or food^83^, rather than visual feedback cues as utilized on the cWCST (i.e., “REPEAT or “SWITCH”). However, what features of a stimulus constitutes it to be a reward or a punishment remains debatable^84^. A comprehensive definition of reward and punishment refers to the behavior that a stimulus induces. That is, a stimulus, which increases (or decreases) the frequency of a preceding action, constitutes a reward (or a punishment)^84^. Feedback cues on the cWCST fall within that definition of reward and punishment, as feedback cues elicit the repetition or avoidance of an action, i.e., the application of a sorting category or the execution of a response (see Fig. 1). Although the interpretation of WCST-feedback cues as reinforcement dates back to initial WCST studies^15^, this interpretation of WCST-feedback cues remains debatable. Alternatively, feedback cues on the cWCST could also be understood in an instruction-based-learning framework^85^. That is, WCST-feedback cues might rather constitute instructions to repeat or switch the previously applied category or executed response than reward or punishment for the application of a category or the execution of a response.

Comparing individual learning rate parameters of the wP-RL model under consideration of the weighting parameter reveals a much stronger impact of MB- than of MF-reinforcement learning on card sorting performance. That is, integrated feedback expectations were stronger driven by MB- than by MF-reinforcement learning. Please note that this finding also holds true when comparing learning rate parameters of the P-RL model, which provided better parameter recovery (see Table S10). The stronger MB-reinforcement learning was no surprise, given the WCST task instructions, which highlight the importance of category-level learning. This finding appears to be rather unusual in comparison to previous studies of MB- and MF-reinforcement learning, which report a more balanced impact of MB- and MF-reinforcement learning on performance^72,86,87^. However, the comparison of parameter estimates in this study to those of other studies of MB- and MF-reinforcement learning is not straightforward due to substantial differences between the cWCST and cognitive paradigms that are specifically designed to study MB- and MF-reinforcement learning, such as multistep decision tasks^72,86,87^. Moreover, further studies are necessary to address the validity of MB- and MF-reinforcement learning as instantiations of category- and response-level learning on the cWCST. However, our results suggest that MB- and MF-reinforcement learning provides a computational framework that accounts for a number of behavioral effects on the cWCST (i.e., the generally higher perseveration propensity than set-loss propensity and the modulation of perseveration propensity by response demands).

Estimates of the MF-learning rate after negative feedback were small when compared to learning rates of MB-reinforcement learning but substantially different from zero. In contrast, estimates of the MF-learning rate after positive feedback were close to zero, indicating that feedback expectations of MF-reinforcement learning were not updated after positive feedback.

The exclusive updating of MF-feedback expectations following negative feedback might be accounted for by the hypothesis of an uncertainty modulated weighting of MB- and MF-reinforcement learning^61^. On card sorting tasks, such as the cWCST, participants face uncertainty about the prevailing sorting category^88–90^. The reception of positive feedback allows identifying the correct sorting category unambiguously, and, by way of this, identifying the response that yields a positive feedback on the upcoming trial (conditional upon a repetition of the sorting category). Thus, following positive feedback, MB-reinforcement learning of categories is associated with low uncertainty. The certainty that occurs under these circumstances may render additional MF-reinforcement learning needless. In contrast, the reception of negative feedback indicates that the application of a category was incorrect. Under these circumstances, the correct category remains uncertain (e.g., negative feedback following the application of the color category indicates that either the shape or number category is correct). On these trials, two responses remain viable for positive feedback, and under these circumstances participants might favor the response that did not produce negative feedback on the previous trial. In sum, response-related MF-reinforcement learning may provide additional guidance for card sorting when MB-reinforcement learning is faced with high uncertainty about the upcoming feedback. It remains to propose an adequate computational description of the uncertainty modulated weighting of MB- and MF-reinforcement learning. Such a description might be based on Bayesian reinforcement-learning algorithms^61,91^, which explicitly quantify the uncertainty about feedback expectations.

We assumed separate learning rates after positive and negative feedback for the reinforcement-learning models. However, previous studies of the AU model were inconclusive as to whether model configurations with separate sensitivity parameters (as a counterpart to learning rate parameters in reinforcement learning) for positive and negative feedback outperform model configurations with a single sensitivity parameter^31,35^. In the present study, parameter estimates of the reinforcement-learning models showed substantial differences between learning rates after positive and negative feedback for MB- and MF-reinforcement learning. These findings suggest that separate learning rates are more appropriate for the studied reinforcement-learning models. This conclusion needs to be further examined by directly comparing the performance of reinforcement-learning models with separate and single learning rates. An alternative approach to separating learning rates by feedback type is the dynamic adjustment of learning rate parameters from trial-to-trial. Such algorithms were proposed long time ago in the context of associative learning^92,93^.

The parallel reinforcement-learning models allow disentangling inertia of MB-feedback expectations from that of MF-feedback expectations. The obtained estimates for inertia parameters of MB- and MF-reinforcement learning were roughly equal. Thus, configurations of the parallel reinforcement-learning models with a single inertia parameter may perform as good as configurations with separate inertia parameters. Future research might address the pooling of MB- and MF-inertia parameters that could provide an appropriate way to reduce complexity of the parallel reinforcement-learning models.

All reinforcement-learning models outperformed the state-of-the-art AU model^31^, indicating that reinforcement learning provides an even more suitable framework than AU for modeling of card sorting performance. This finding held true even when we compared models with equal complexity (i.e., both the MB-RL model and the AU model incorporated four individual parameters). The frameworks of AU and reinforcement learning as implemented in this study differ with regard to four major aspects. First, the AU framework assumes fixed updating of attentional category prioritizations from trial-to-trial (given by individual parameters $p^{ + }$ and $p^{ - }$). In contrast, reinforcement learning assumes that updating of feedback-expectation is a function of individual learning rates and prediction errors: larger prediction errors, which are scaled by learning rates, are associated with a stronger updating of feedback expectations^54^. Second, the AU framework assumes that attentional prioritizations of all categories are updated on any trial (e.g., after positive feedback, the attentional category prioritization of the applied category increases, and all other prioritizations decrease), whereas reinforcement learning updates only feedback expectations of the applied category and/or of the executed response. Third, in order to derive response probabilities, the AU framework incorporates an algorithm that divides each attentional category prioritization by the overall sum of attentional category prioritizations. In contrast, we assumed a “softmax” rule to derive response probabilities for reinforcement-learning models, which is based on the exponential function. Finally, reinforcement learning as utilized in this study incorporates inertia of feedback expectations.

Our results suggest that feedback-driven learning, as exemplified by card sorting performance, can be conceptualized as two parallel yet independent reinforcement learning processes^61,87,94^. These learning processes differ with regard to their level of abstraction. A cognitive learning process, which may also be described as goal-directed or executive^78,95,96^, operates at an abstract level to guide task-appropriate actions (i.e., formalized as MB-reinforcement learning in this study). When task demands change (e.g., indicated by a negative feedback on the cWCST), and uncertainty about feedback expectations of the cognitive learning process is high, a behavioral learning process complements the cognitive learning process^61^. The behavioral learning process was formalized as MF-reinforcement learning in this study. It may be described as habitual^95,96^, because it bypasses the abstract level by simply favoring actions that were followed by positive feedback, and by avoiding actions that were followed by negative feedback. Parallel cognitive and behavioral reinforcement learning processes seem to complement each other; in particular when the cognitive learning system is faced with uncertainty about feedback expectations.

## Conclusions

We presented a detailed evaluation of computational models of card sorting performance in a large sample of healthy volunteers (*N* = 375). We proposed that valid computational models of card sorting performance should be able to account for a wide range of behavioral effects that are detectable on card sorting tasks, such as the cWCST. Hence, a benchmark for all model comparisons in this study was not only the recovery of traditional perseveration and set-loss error propensities. In addition, all computational models were evaluated by their ability to recover the recently reported^50^ modulation of perseveration propensities by response demands, which we successfully replicated in the present study. Against this background, parallel reinforcement-learning models, which incorporate MB- and MF-reinforcement learning, should be considered as valid computational models of card sorting performance. However, a more fine-grained analysis of individual model performance suggests that not all participants are best described by parallel MB- and MF-reinforcement learning.

In conclusion, parallel reinforcement-learning models provide a new theoretical perspective on card sorting by conceptualizing WCST performance as parallel MB- and MF-reinforcement learning. Our computational approach offers a novel framework to discern individual differences in latent processes of behavioral flexibility in healthy and patient populations.

## Methods

### Data collection

#### Participants

A total of *N* = 407 participants (155 male, two preferred not to say; *M* = 23.47 years; *SD* = 4.83 years) completed the cWCST. We excluded 32 participants due to invalid test performance, resulting in a final sample of *N* = 375 participants (144 male, one preferred not to say; *M* = 23.17 years; *SD* = 4.37 years). Test performance was considered as invalid when one of the three categories was more or less frequently applied than the overall mean of applications of that category plus/ minus three standard deviations. The studied data were originally published by Lange and Dewitte^53^. The study was approved by the local ethics committee of the KU Leuven (G-2016 12 694). All participants gave informed consent in accordance with the Declaration of Helsinki.

#### Computerized Wisconsin Card Sorting Test

The cWCST^12,35,53,68^ requires participants to match stimulus cards according to one of three possible categories. Stimulus cards varied on three dimensions that equaled the three viable categories *U* = {color, form, number}. Participants indicated their response by pressing one of four keys *V* = {response 1, response 2, response 3, response 4} that were spatially mapped to the position of the key cards *W* = {one red triangle, two green stars, three yellow crosses, and four blue balls}. The 24 stimulus cards shared not more than one dimension with the same key card, rendering the applied category unambiguously identifiable. Responses were followed by a positive or negative visual feedback cue (“REPEAT” or “SWITCH”, respectively)^89^. On any trial, the application of the correct category led to the presentation of a positive feedback cue (*m* = 50.84% of trials, *SD* = 7.20%), whereas the application of all other sorting categories or the selection of the key card that matched none of the viable sorting categories, led to the presentation of a negative feedback cue (*m* = 49.16% of trials, *SD* = 7.20%). Correct categories changed in an unpredictable manner after runs of two or more correct category repetitions (average number of correct category repetitions to trigger a switch of the correct sorting category = 3.5). Participants were required to complete 41 switches of the correct category, with a maximum of 250 trials to complete these 41 switches of the correct category and a practice session including 6 switches of the correct sorting category. Prior to the experimental session, participants were explicitly informed about the three possible sorting categories and about the fact that the correct category would switch from time to time. For all analyses, we excluded trials with responses that matched no viable sorting category; as such rarely occurring events (0.54% of all trials) would cause errors in parameter estimation. Parameter estimation in this study is based on assigning logarithmized probabilities to participants’ responses using a computational model. However, the AU model assigns a probability of zero to responses that match no viable category, which makes the corresponding logarithm undefined. For further details on the cWCST, see Lange and Dewitte^53^.

### Behavioral analysis

Conditional error probabilities were analyzed using JASP version 0.10^97^. Default settings of JASP were used for the Bayesian repeated measures ANOVA with uniform prior probabilities for all ANOVA models under consideration (P(M) = 0.2). In addition to posterior probabilities, we report logarithmized Bayes factors for an ANOVA model when compared to all the other ANOVA models under consideration^98^. Note that we did not analyze the number of completed categories, as this dependent variable is fixed on the cWCST.

### Computational modeling

The wP-RL and the P-RL models incorporated MB- and MF-reinforcement learning. In previous approaches of MB-reinforcement learning, participants operate on an abstract level, which incorporates feedback expectations for the prevailing task state (i.e., the correct sorting category) and a transition structure of task states (i.e., when categories will switch and which category will be correct)^87^. However, as switches of the correct category on the cWCST are supposed to be unpredictable, participants cannot learn its transition structure. Following, we assume that the abstract cognitive model of MB-reinforcement learning reduces to trial-by-trial learning of feedback expectations for the application of categories.

Individual parameters of the wP-RL and the P-RL models are learning rate parameters for MB- and MF-reinforcement learning, which were further differentiated by feedback type. Learning rates give the extent to which prediction errors are integrated into feedback expectations following positive or negative feedback. Highest values of learning rates indicate that a prediction error will be added to the feedback expectations of the applied category or the executed response without attenuation. In contrast, with the lowest possible learning rate, no updating of the feedback expectation of the applied category or the executed response will happen. In addition, MB- and MF-inertia parameters, which quantify how much information from previous trials will be retained for the current trial. With highest values of inertia parameters, feedback expectations from the previous trial will transfer to the current trial without mitigation. In contrast, with lowest values of inertia parameters, feedback expectations are not transferred to the current trial. In such cases, responding depends entirely on the last received feedback. Thus, learning rate parameters and inertia parameters represent distinct model mechanisms^70,73^, i.e., the strength of feedback integration into feedback expectations of the applied category or the executed response and the trial-to-trial inertia of all feedback expectations, respectively. Lastly, an individual temperature parameter gives the extent to which responding accords to integrated feedback expectations. More precisely, the temperature parameter indicates whether differences in integrated feedback expectations are attenuated (temperature values higher than 1) or emphasized (temperature values less than 1). The wP-RL model incorporates an additional weighting parameter, which quantifies the relative strength of MB- over MF-reinforcement learning. High configurations of the weighting parameter (weighting values higher than 0.5) indicate a stronger weighting of MB- over MB-reinforcement learning and vice versa.

#### Model-based reinforcement learning

The implemented MB-reinforcement-learning algorithm operates on an abstract level, which is represented by a 3 (categories) × 1 vector **Q**_*C*_(*t*). **Q**_*C*_(*t*) quantifies the feedback expectation for the application of any category on trial *t*. Inertia of feedback expectations from one trial to the next is modeled as:

1 $${\mathbf{Q}}_{C}^{^{\prime}} (t) = \gamma_{MB} *{\mathbf{Q}}_{C} (t)$$

where $\gamma_{MB}$ gives the strength of inertia. $\gamma_{MB}$ ranges from 0 to 1, with high values representing higher inertia of feedback expectations. Next, trial-wise prediction errors $\delta_{MB} (t)$ are computed with regard to the category *u ϵ U*, which has been applied on trial *t*, as:

2 $$\delta_{MB} (t) = r(t) - Q_{C,u}^{^{\prime}} (t)$$

where *r*(*t*) is 1 for positive and − 1 for negative feedback. Feedback expectations of categories are updated by a delta-learning rule:

3 $${\mathbf{Q}}_{C} (t + 1) = {\mathbf{Q}}_{C}^{^{\prime}} (t) + {\mathbf{Z}}_{C} (t)*\alpha_{MB} *\delta_{MB} (t)$$

where **Z**_*C*_(*t*) is a 3 × 1 dummy vector, which is 1 for the applied category *u* and 0 for all other categories on trial *t*. **Z**_*C*_(*t*) ensured that only the expected feedback value of the applied category is updated in response to the prediction error. In line with existing reinforcement-learning models^99–102^ and the state-of-the-art AU model of card sorting performance^31^, we assumed distinct learning rate parameters for positive and negative feedback, $\alpha_{MB}^{ + }$ and $\alpha_{MB}^{ - }$, which quantify the degree to which prediction errors are integrated into current feedback expectations. Learning rates range from 0 to 1.

Lastly, feedback expectations for the application of categories **Q**_*C*_(*t*) are assigned to responses. More precisely, MB-feedback expectations of responses are represented by a 4 (responses) × 1 vector **Q**_*MB*_(*t*). For response *v* ϵ *V* on trial *t,* **Q**_*MB*_(*t*) is computed as:

4 $$Q_{MB,v} (t) = {\mathbf{X}}_{v}^{{\text{T}}} (t){ }{\mathbf{Q}}_{C} (t)$$

with ${\mathbf{X}}_{v} (t)$ is a 3 (categories) × 1 vector that represents the match between a stimulus card and key card *w* (corresponding to response *v*) on trial *t* with regard to the color, form, and number category. Here, 1 indicates a match and 0 indicates no match. ${\mathbf{X}}_{v}^{{\text{T}}} (t)$ denotes the transpose of ${\mathbf{X}}_{v} (t)$. In order to account for responses that match no viable sorting category (i.e., certainly yield a negative feedback with regard to MB-reinforcement learning), we assigned these responses a MB-feedback expectation of − 1. Therefore, ${\mathbf{X}}_{v}^{{\text{T}}} (t){ }{\mathbf{Q}}_{C} (t)$ in Eq. (4) was set to − 1, if key card *v* on trial *t* matches none of the valid sorting categories.

#### Model-free reinforcement learning

MF-reinforcement learning operates directly on feedback expectations of responses. MF-reinforcement learning is based on a 4 (responses) × 1 vector **Q**_*MF*_(*t*), which gives feedback expectations for the execution of any response on trial *t*. First, the inertia of **Q**_*MF*_(*t*) is computed as:

5 $${\mathbf{Q}}_{MF}^{^{\prime}} (t) = \gamma_{MF} *{\mathbf{Q}}_{MF} (t)$$

where $\gamma_{MF}$ modulates the strength of inertia. Trial-wise prediction errors of MF-reinforcement learning are computed with regard to the executed response *v *∈ *V* on trial *t* as:

6 $$\delta_{MF} (t) = r(t) - Q_{MF,v}^{^{\prime}} (t)$$

Next, feedback expectations are updated as:

7 $${\mathbf{Q}}_{MF} (t + 1) = {\mathbf{Q}}_{MF}^{^{\prime}} (t) + {\mathbf{Z}}_{MF} (t)*\alpha_{MF} *\delta_{MF} (t)$$

where **Z**_*MF*_(*t*) is a 4 × 1 dummy vector that is 1 for the executed response *v* and 0 for all other responses on trial *t*, which, again, ensured that only feedback expectations of the executed response are updated in response to the prediction error. We assumed different learning rate parameters for positive and negative feedback, $\alpha_{MF}^{ + }$ and $\alpha_{MF}^{ - }$, respectively.

#### Integration and response probabilities

In order to compute response probabilities, MB- and MF-feedback expectations are integrated. For the P-RL model, the integrated feedback expectation on trial *t* ${\varvec{Q}}_{sum} (t)$ is computed as:

8 $${\varvec{Q}}_{sum} (t) = {\mathbf{Q}}_{MB} (t) + {\varvec{Q}}_{MF} (t)$$

In contrast, the wP-RL model incorporates an additional weighting parameter that modulates the integration of MB- and MF-feedback expectations as:

9 $${\varvec{Q}}_{sum} (t) = w*{\mathbf{Q}}_{MB} (t) + (1-w)*{\user2{Q}}_{MF} (t)$$

with the weighting parameter ranged from 0 to 1.

Finally, the probability of executing response *v* on trial *t* is computed using a “softmax” logistic function on integrated feedback expectations as:

10 $$P_{v} (t) = \frac{{e^{{\frac{{Q_{sum,v} (t)}}{\tau }}} }}{{\mathop \sum \nolimits_{j = 1}^{4} e^{{\frac{{Q_{sum,j} (t)}}{\tau }}} }}$$

with $\tau \in {\mathcal{R}}^{ + }$ that is an temperature parameter indicating whether differences in integrated feedback expectations are attenuated $(\tau > 1)$ or emphasized $(0 < \tau < 1)$.

#### Attentional-updating model

The AU model^31^ operates on a 3 (categories) × 1 vector ${\mathbf{a}}(t)$ that quantifies attentional category prioritizations on any trial *t* as:

11 $${\mathbf{a}}(t) = \left( {\begin{array}{*{20}c} {a_{color} (t)} \\ {a_{shape} (t)} \\ {a_{number} (t)} \\ \end{array} } \right)$$

${\mathbf{a}}(t)$ is trial-wise updated based on a feedback signal. The feedback signal ${\varvec{s}}(t)$ is computed as a function of current attentional category prioritizations, feedback, and an individual attentional focus parameter $f \in {\mathcal{R}}^{ + }$. More precisely, on positive feedback trials, ${\varvec{s}}(t)$ of category *u* is given by:

12 $$s(t)_{u} |positive = \frac{{m_{v,u} (t)a_{u}^{{}} (t)^{f} }}{{\mathop \sum \nolimits_{h = 1}^{3} m_{v,h} (t)a_{h}^{{}} (t)^{f} }}$$

and on negative feedback trials by:

13 $$s(t)_{u} |negative = \frac{{\left( {1 - m_{v,u} (t)} \right)a_{u}^{{}} (t)^{f} }}{{\mathop \sum \nolimits_{h = 1}^{3} \left( {1 - m_{v,h} (t)} \right)a_{h}^{{}} (t)^{f} }}$$

${\mathbf{m}}_{v} (t)$ is a 3 (categories) × 1 vector representing matches between a category *u* and the selected key card *w* (corresponding to response *v*) on trial *t*. Let ${\mathbf{m}}_{v,u} (t)$ be 1 for a match and 0 otherwise. The individual attentional focus $f$ either emphasizes or equalizes differences in the feedback signal.

Attentional category prioritizations for the next trial ${\mathbf{a}}(t + 1)$ are updated by integrating feedback from the current trial:

14 $${\mathbf{a}}(t + 1) = (1 - p)*{\mathbf{a}}(t) + p*{\mathbf{s}}(t)$$

Here, the ratio of information integrated from the previous trial and the current feedback signal is given by the individual parameter $p$ ranging from 0 to 1. The implemented configuration of the AU model is based on separate $p$ parameters for positive and negative feedback, $p^{ + }$ and $p^{ - }$, respectively. The probability of response *v* on trial *t* is then given by:

15 $$P_{v} (t) = \frac{{{\varvec{m}}_{v}^{{\text{T}}} (t){\mathbf{a}}(t)^{d} }}{{\mathop \sum \nolimits_{j = 1}^{4} \left( {{\varvec{m}}_{j}^{{\text{T}}} (t){\mathbf{a}}(t)^{d} } \right)}}$$

with T denotes the transpose of ${\mathbf{m}}_{v} (t)$. Here, $d \in {\mathcal{R}}^{ + }$ represents participant’s decision consistency that either renders responses more deterministic or random.

#### Model space

We considered four computational models of card sorting performance. First, we implemented the wP-RL model incorporating MB- and MF-reinforcement learning weighted by an individual *w* parameter as described above. Second, we considered the P-RL model incorporating MB- and MF-reinforcement learning but no weighting parameter. Third, we implemented the MB-RL model that only operates on MB-reinforcement learning, i.e., trial-by-trial updating of feedback expectations accorded to Eqs. (1) – (4) and response probabilities were computed by adapting the “softmax” rule (Eq. 10) on ${\mathbf{Q}}_{MB} (t)$. Note that we did not implement a model that incorporates MF-reinforcement learning only, as it is psychologically implausible with regard to efficient card sorting performance. In order to test whether inertia parameters increase performance of the reinforcement-learning models, we also fitted simpler configurations of the wP-RL, the P-RL, and the MB-RL model without inertia parameters (i.e., fixing $\gamma_{MB}$ and $\gamma_{MF}$ to 1). However, *K*-fold cross validation revealed that model configurations with fixed inertia parameters did not perform better than configurations with free-to-vary inertia parameters (Δelpd between the wP-RL model with and without inertia parameters = − 6,672; *SE* = 173; Δelpd between the P-RL model with and without inertia parameters = − 13,979; *SE* = 197; Δelpd between the MB-RL model with and without inertia parameters = − 13,814; *SE* = 208). Thus, inertia parameters significantly improved model performance of the wP-RL, the P-RL, and the MB-RL model. Lastly, we implemented the state-of-the art AU model^31^. Note that we used a full configuration of the AU model with all four individual parameters set free to vary. We also considered an AU model configuration with reduced complexity (i.e., the number of individual parameters): We implemented a configuration with fixed attentional focus parameter (*f* = 1), which was reported as best-performing^31^. As hierarchical Bayesian analysis failed for this model configuration and *p*^+^ seemed to converge to 1, we also fixed *p*^+^ to 0.9999. However, *K*-fold cross validation revealed that the full model outperformed the reduced configuration. Note that the reduced model configuration was not able to simulate the finding of an error modulation by response demands. See https://osf.io/9te5u/ for results and further details.

#### Parameter estimation

We used hierarchical Bayesian analysis^101,103–108^ for individual parameter estimation by means of RStan^109^. To increase efficiency of parameter estimation, we implemented non-centered parameterizations and conducted parameter estimation in an unconstrained space^101,103,110^. For example, the learning rate following positive feedback of MB-reinforcement learning, $\alpha_{MB}^{ + }$, was formally specified by a vector of individual-level parameters as:

16 $${{\varvec{\alpha}}}_{MB}^{ + } = Probit\left( {\mu_{{\alpha_{MB}^{ + } }} + \sigma_{{\alpha_{MB}^{ + } }} *{{\varvec{\upalpha}}}_{{MB}}^{\prime + } } \right)$$

Individual MB-learning rate parameters following positive feedback, $\alpha_{MB}^{ + } ,$ were given by group-level location and scale parameters, $\mu_{{\alpha_{MB}^{ + } }}$ and $\sigma_{{\alpha_{MB}^{ + } }}$, respectively, and a vector of individual-level location parameters, ${{\varvec{\alpha}}}_{MB}^{ \prime+ }$. The parameters $\mu_{{\alpha_{MB}^{ + } }}$, $\sigma_{{\alpha_{MB}^{ + } }}$, and ${{\varvec{\alpha}}}_{MB}^{ \prime+ }$ were estimated in an unconstrained space (i.e., [− ∞, ∞]) and their linear combination was Probit-transformed to a constrained space. The Probit is the inverse-cumulative distribution of the standard normal distribution, mapping unconstrained values to the interval ]0,1[. For model parameters that had no upper boundaries (e.g., the temperature parameter $\tau$ could exceed 1), we scaled Eq. (16) by multiplying it with five^103^. In line with previous studies using hierarchical Bayesian analysis^101,103^, we assumed that group-level location parameters had normal prior distributions (*μ* = 0, *σ* = 1) and Cauchy prior distributions for scale parameters (*μ* = 0, *σ* = 5). For individual-level location parameters, we implemented normal prior distributions (*μ* = 0, *σ* = 1).

For parameter estimation, we initialized Q-values of the wP-RL, the P-RL, and the MB-RL models as 0. As suggested by Bishara et al.^31^, values of **a** were initialized as 1/3. Sampling was done using three chains including 1,000 iterations and 500 warm-up iterations each. Convergence of chains was checked visually by trace-plots and quantitatively by the $\hat{R}$ statistic^111^. The implemented code was adapted from the R package hBayesDM^103^ and can be downloaded from https://osf.io/9te5u/, which also provides further specifications of the utilized sampling algorithm.

#### Relative model performance

In order to adjudicate between computational models, their performance needs to be quantified on a scale that is comparable across computational models. Such a performance quantification is achieved by, for example, assessing a model’s predictive accuracy^112^. The major challenge in quantifying a model’s performance is to account for the trade-off between a model’s complexity and its goodness-of-fit. A model with an unnecessary high number of degrees of freedom will present a good fit to the data. However, such a model is not parsimonious and will perform poorly when it comes to predict novel data, as the additional degrees of freedom fit idiosyncratic, nonreplicable noise. In contrast, a too parsimonious model might not show the necessary complexity to present a good fit to the data, thus decreasing its goodness of prediction of novel data^112^. Many methods have been proposed in order to account for the complexity-fit tradeoff, like the AIC^113^ and the BIC^114^, which are based on the assumption that complexity can be unambiguously quantified (i.e., by the number of a model’s individual parameters). In contrast, cross-validation methods assess a models predictive accuracy directly by fitting a model to training data and testing its performance on validation data.

In this study, we used *K*-fold cross validation following the procedure outlined by Vehtari, Gelman, and Gabry^115^. Participants were randomly assigned to *K* = 5 subsets $y_{k}$. Computational models were fitted separately to each training set $y_{{\left( { - k} \right)}}$, including all data but subset $y_{k}$. Next, we used parameter estimates of training set $y_{{\left( { - k} \right)}}$ to compute the predicted probabilities of responses in $y_{k}$. For any participant, the product of predicted response probabilities across all trials was averaged across iterations of parameter estimation and logarithmized, which gives the elpd. The sum of elpd values over all participants was used as a metric for a models group-level predictive accuracy.

Relative model performance was quantified by the difference in elpd between the model with the lowest absolute elpd and any other model (Δelpd). The lower the absolute elpd, the better is a model’s performance (i.e., a better predictive accuracy). Hence, larger absolute Δelpd-values indicate worse model performance. We also report standard errors associated with the Δelpd-values. Note that we chose *K* = 5 for reasons of computation time. The code used for *K*-fold cross validation was adapted from Nicenboim and Vasishth^116^.

#### Absolute model performance

For assessment of absolute model performance, we used the post-hoc absolute fit method^73^. The post-hoc absolute fit method conducts one-trial-ahead predictions of individual responses on trial *t*, using estimated individual model parameters as well as observed responses and received feedback on all preceding trials. More precisely, for any participant, model parameters were randomly drawn from the individual-level posterior distribution of model parameters. Next, a participant’s response on trial *t* was simulated by informing the computational model of interest with estimated parameters as well as responses and feedback history from trial 1 to *t*-1. For any participant and across all trials, conditional error probabilities were computed based on simulated responses as described for behavioral data. This procedure was repeated for 1,000 iterations. For any participant, conditional error probabilities were averaged over all iterations of the procedure and entered into a Bayesian repeated measures ANOVA. Additionally, as an indicator of a model’s ability to account for inter-individual variance of behavioral performance indices, we computed the *R*^2^ statistic (i.e., inter-individual variance accounted for by a model divided by the variance of the observed data) using Bayesian linear regression of observed conditional error probabilities when predicted by simulated conditional error probabilities by means of JASP. For a detailed account of the post-hoc absolute fit method, see Steingroever et al.^73^.

## Supplementary information

Supplementary Information.

## Data Availability

All data and code are available at https://osf.io/9te5u/.

## References

[1] Diamond A (2013). Executive functions. Annu. Rev. Psychol..

[2] Braem S, Egner T (2018). Getting a grip on cognitive flexibility. Curr. Dir. Psychol. Sci..

[3] Miyake A (2000). The unity and diversity of executive functions and their contributions to complex ‘frontal lobe’ tasks: a latent variable analysis. Cogn. Psychol..

[4] Badre D, Wagner AD (2006). Computational and neurobiological mechanisms underlying cognitive flexibility. Proc. Natl. Acad. Sci..

[5] Allport DA, Styles EA, Hsieh S, Umiltà C, Moscovitch M (1994). Shifting intentional set: exploring the dynamic control of tasks. Attention and Performance Series. Attention and Performance 15: Conscious and Nonconscious Information Processing.

[6] Grange JA, Houghton G (2014). Task Switching and Cognitive Control.

[7] Kiesel A (2010). Control and interference in task switching—a review. Psychol. Bull..

[8] Rogers RD, Monsell S (1995). Costs of a predictible switch between simple cognitive tasks. J. Exp. Psychol. Gen..

[9] Geurts HM, Corbett B, Solomon M (2009). The paradox of cognitive flexibility in autism. Trends Cogn. Sci..

[10] Hommel B, Colzato LS (2017). The social transmission of metacontrol policies: mechanisms underlying the interpersonal transfer of persistence and flexibility. Neurosci. Biobehav. Rev..

[11] Lange F (2016). Meta-analytical and electrophysiological evidence for executive dysfunction in primary dystonia. Cortex.

[12] Lange F, Seer C, Kopp B (2017). Cognitive flexibility in neurological disorders: cognitive components and event-related potentials. Neurosci. Biobehav. Rev..

[13] Meiran N, Diamond GM, Toder D, Nemets B (2011). Cognitive rigidity in unipolar depression and obsessive compulsive disorder: examination of task switching, Stroop, working memory updating and post-conflict adaptation. Psychiatry Res..

[14] Berg EA (1948). A simple objective technique for measuring flexibility in thinking. J. Gen. Psychol..

[15] Grant DA, Berg EA (1948). A behavioral analysis of degree of reinforcement and ease of shifting to new responses in a Weigl-type card-sorting problem. J. Exp. Psychol..

[16] Heaton RK, Chelune GJ, Talley JL, Kay GG, Curtiss G (1993). Wisconsin Card Sorting Test Manual: Revised and Expanded.

[17] Nelson HE (1976). A modified card sorting test sensitive to frontal lobe defects. Cortex.

[18] Beeldman E (2016). The cognitive profile of ALS: a systematic review and meta-analysis update. J. Neurol. Neurosurg. Psychiatry.

[19] Demakis GJ (2003). A meta-analytic review of the sensitivity of the Wisconsin Card Sorting Test to frontal and lateralized frontal brain damage. Neuropsychology.

[20] Dirnberger G, Jahanshahi M (2013). Executive dysfunction in Parkinson’s disease: a review. J. Neuropsychol..

[21] Kudlicka A, Clare L, Hindle JV (2011). Executive functions in Parkinson’s disease: systematic review and meta-analysis. Mov. Disord..

[22] Lange F, Brückner C, Knebel A, Seer C, Kopp B (2018). Executive dysfunction in Parkinson’s disease: a meta-analysis on the Wisconsin Card Sorting Test literature. Neurosci. Biobehav. Rev..

[23] Lange F, Seer C, Müller-Vahl K, Kopp B (2017). Cognitive flexibility and its electrophysiological correlates in Gilles de la Tourette syndrome. Dev. Cogn. Neurosci..

[24] Lange F (2016). Impaired set-shifting in amyotrophic lateral sclerosis: an event-related potential study of executive function. Neuropsychology.

[25] Nyhus E, Barceló F (2009). The Wisconsin Card Sorting Test and the cognitive assessment of prefrontal executive functions: a critical update. Brain Cogn..

[26] Roberts ME, Tchanturia K, Stahl D, Southgate L, Treasure J (2007). A systematic review and meta-analysis of set-shifting ability in eating disorders. Psychol. Med..

[27] Romine C (2004). Wisconsin Card Sorting Test with children: a meta-analytic study of sensitivity and specificity. Arch. Clin. Neuropsychol..

[28] Shin NY, Lee TY, Kim E, Kwon JS (2014). Cognitive functioning in obsessive-compulsive disorder: a meta-analysis. Psychol. Med..

[29] Snyder HR (2013). Major depressive disorder is associated with broad impairments on neuropsychological measures of executive function: a meta-analysis and review. Psychol. Bull..

[30] Milner B (1963). Effects of different brain lesions on card sorting. Arch. Neurol..

[31] Bishara AJ (2010). Sequential learning models for the Wisconsin card sort task: assessing processes in substance dependent individuals. J. Math. Psychol..

[32] Buchsbaum BR, Greer S, Chang WL, Berman KF (2005). Meta-analysis of neuroimaging studies of the Wisconsin Card-Sorting task and component processes. Hum. Brain Mapp..

[33] Dehaene S, Changeux JP (1991). The Wisconsin Card Sorting Test: theoretical analysis and modeling in a neuronal network. Cereb. Cortex.

[34] Ridderinkhof KR, Span MM, van der Molen MW (2002). Perseverative behavior and adaptive control in older adults: performance monitoring, rule induction, and set shifting. Brain Cogn..

[35] Steinke A, Lange F, Seer C, Kopp B (2018). Toward a computational cognitive neuropsychology of Wisconsin card sorts: a showcase study in Parkinson’s disease. Comput. Brain Behav..

[36] Botvinick MM, Cohen JD (2014). The computational and neural basis of cognitive control: charted territory and new frontiers. Cogn. Sci..

[37] Oberauer K, Lewandowsky S (2019). Addressing the theory crisis in psychology. Psychon. Bull. Rev..

[38] Granato, G. & Baldassarre, G. Goal-directed top-down control of perceptual representations: a computational model of the Wisconsin Card Sorting Test. In *2019 Conference on Cognitive Computational Neuroscience* (Cognitive Computational Neuroscience, 2019). 10.32470/CCN.2019.1168-0

[39] Amos A (2000). A computational model of information processing in the frontal cortex and basal ganglia. J. Cogn. Neurosci..

[40] Berdia S, Metz JT (1998). An artificial neural network stimulating performance of normal subjects and schizophrenics on the Wisconsin card sorting test. Artif. Intell. Med..

[41] Kaplan GB, Şengör NS, Gürvit H, Genç İ, Güzeliş C (2006). A composite neural network model for perseveration and distractibility in the Wisconsin card sorting test. Neural Netw..

[42] Kimberg DY, Farah MJ (1993). A unified account of cognitive impairments following frontal lobe damage: the role of working memory in complex, organized behavior. J. Exp. Psychol. Gen..

[43] Levine DS, Prueitt PS (1989). Modeling some effects of frontal lobe damage—novelty and perseveration. Neural Netw..

[44] Caso A, Cooper RP, Gunzelmann G, Howes A, Tenbrink T, Davelaar EJ (2017). A model of cognitive control in the Wisconsin card sorting test: integrating schema theory and basal ganglia function. Proceedings of the 39th Annual Conference of the Cognitive Science Society.

[45] Hazy TE, Frank MJ, O’Reilly RC (2007). Towards an executive without a homunculus: computational models of the prefrontal cortex/basal ganglia system. Philos. Trans. R. Soc. B Biol. Sci..

[46] Williams CC, Hassall CD, Lindenbach T, Krigolson OE (2019). Reward prediction errors reflect an underlying learning process that parallels behavioural adaptations: a trial-to-trial analysis. Comput. Brain Behav..

[47] Cella M (2014). Identifying cognitive remediation change through computational modelling—effects on reinforcement learning in schizophrenia. Schizophr. Bull..

[48] Farreny A (2016). Study of positive and negative feedback sensitivity in psychosis using the Wisconsin Card Sorting Test. Compr. Psychiatry.

[49] Gläscher J, Adolphs R, Tranel D (2019). Model-based lesion mapping of cognitive control using the Wisconsin Card Sorting Test. Nat. Commun..

[50] Kopp B, Steinke A, Bertram M, Skripuletz T, Lange F (2019). Multiple levels of control processes for Wisconsin Card Sorts: an observational study. Brain Sci..

[51] Steinke, A., Lange, F. & Kopp, B. A multi-level reinforcement-learning model of Wisconsin Card Sorting Test performance. In *2019 Conference on Cognitive Computational Neuroscience* (2019). 10.32470/CCN.2019.1030-0

[52] Barceló F (2003). The Madrid card sorting test (MCST): a task switching paradigm to study executive attention with event-related potentials. Brain Res. Protoc..

[53] Lange F, Dewitte S (2019). Cognitive flexibility and pro-environmental behaviour: a multimethod approach. Eur. J. Pers..

[54] Sutton RS, Barto AG (1998). Reinforcement Learning: An Introduction.

[55] Niv Y (2009). Reinforcement learning in the brain. J. Math. Psychol..

[56] Silvetti M, Verguts T, Bright P (2012). Reinforcement learning, high-level cognition, and the human brain. Neuroimaging—Cognitive and Clinical Neuroscience.

[57] Gerraty RT (2018). Dynamic flexibility in striatal-cortical circuits supports reinforcement learning. J. Neurosci..

[58] Fontanesi L, Gluth S, Spektor MS, Rieskamp J (2019). A reinforcement learning diffusion decision model for value-based decisions. Psychon. Bull. Rev..

[59] Fontanesi L, Palminteri S, Lebreton M (2019). Decomposing the effects of context valence and feedback information on speed and accuracy during reinforcement learning: a meta-analytical approach using diffusion decision modeling. Cogn. Affect. Behav. Neurosci..

[60] Caligiore D, Arbib MA, Miall RC, Baldassarre G (2019). The super-learning hypothesis: integrating learning processes across cortex, cerebellum and basal ganglia. Neurosci. Biobehav. Rev..

[61] Daw ND, Niv Y, Dayan P (2005). Uncertainty-based competition between prefrontal and dorsolateral striatal systems for behavioral control. Nat. Neurosci..

[62] Kool W, Gershman SJ, Cushman FA (2017). Cost-benefit arbitration between multiple reinforcement-learning systems. Psychol. Sci..

[63] Gläscher J, Daw N, Dayan P, O’Doherty JP (2010). States versus rewards: dissociable neural prediction error signals underlying model-based and model-free reinforcement learning. Neuron.

[64] Botvinick MM (2019). Reinforcement learning, fast and slow. Trends Cogn. Sci..

[65] O’Doherty JP, Cockburn J, Pauli WM (2017). Learning, reward, and decision making. Annu. Rev. Psychol..

[66] Verguts T, Egner T (2017). Computational models of cognitive control. The Wiley Handbook of Cognitive Control.

[67] Schretlen DJ (2010). Modified Wisconsin Card Sorting Test (M-WCST): Professional Manual.

[68] Lange F (2016). Decomposing card-sorting performance: effects of working memory load and age-related changes. Neuropsychology.

[69] Palminteri S, Wyart V, Koechlin E (2017). The importance of falsification in computational cognitive modeling. Trends Cogn. Sci..

[70] Erev I, Roth AE (1998). Predicting how people play games: reinforcement learning in experimental games with unique, mixed strategy equilibria. Am. Econ. Rev..

[71] Steingroever H, Wetzels R, Wagenmakers E-J (2013). Validating the PVL-Delta model for the Iowa gambling task. Front. Psychol..

[72] Daw ND, Gershman SJ, Seymour B, Dayan P, Dolan RJ (2011). Model-based influences on humans’ choices and striatal prediction errors. Neuron.

[73] Steingroever H, Wetzels R, Wagenmakers EJ (2014). Absolute performance of reinforcement-learning models for the Iowa Gambling Task. Decision.

[74] Konstantinidis E, Speekenbrink M, Stout JC, Ahn W-Y, Shanks DR (2014). To simulate or not? Comment on Steingroever, Wetzels, and Wagenmakers (2014). Decision.

[75] Artiola-i-Fortuny L, Heaton RK (1996). Standard versus computerized administration of the Wisconsin Card Sorting Test. Clin. Neuropsychol..

[76] Tien AY (1996). Computerized Wisconsin Card Sorting Test: comparison with manual administration. Kaohsiung J. Med. Sci..

[77] Feldstein SN (1999). A comparison of computerized and standard versions of the Wisconsin Card Sorting Test. Clin. Neuropsychol..

[78] Banich MT, Compton RJ (2018). Cognitive Neuroscience.

[79] Steinke A, Lange F, Seer C, Hendel MK, Kopp B (2020). Computational modeling for neuropsychological assessment of bradyphrenia in Parkinson’s disease. J. Clin. Med..

[80] Steinke A, Lange F, Seer C, Petri S, Kopp B (2020). A computational study of executive dysfunction in amyotrophic lateral sclerosis. J. Clin. Med..

[81] Wilson RC, Collins AG (2019). Ten simple rules for the computational modeling of behavioral data. Elife.

[82] Doll BB, Duncan KD, Simon DA, Shohamy D, Daw ND (2015). Model-based choices involve prospective neural activity. Nat. Neurosci..

[83] Schultz W, Dayan P, Montague PR (1997). A neural substrate of prediction and reward. Science.

[84] Schultz W (2015). Neuronal reward and decision signals: from theories to data. Physiol. Rev..

[85] Wolfensteller U, Ruge H (2012). Frontostriatal mechanisms in instruction-based learning as a hallmark of flexible goal-directed behavior. Front. Psychol..

[86] Sharp ME, Foerde K, Daw ND, Shohamy D (2016). Dopamine selectively remediates ‘model-based’ reward learning: a computational approach. Brain.

[87] Moran R, Keramati M, Dayan P, Dolan RJ (2019). Retrospective model-based inference guides model-free credit assignment. Nat. Commun..

[88] Lange F (2018). Effects of rule uncertainty on cognitive flexibility in a card-sorting paradigm. Acta Psychol. (Amst).

[89] Kopp B, Lange F (2013). Electrophysiological indicators of surprise and entropy in dynamic task-switching environments. Front. Hum. Neurosci..

[90] Lange F, Seer C, Finke M, Dengler R, Kopp B (2015). Dual routes to cortical orienting responses: novelty detection and uncertainty reduction. Biol. Psychol..

[91] Kruschke JK (2008). Bayesian approaches to associative learning: from passive to active learning. Learn. Behav..

[92] Mackintosh NJ (1975). A theory of attention: variations in the associability of stimuli with reinforcement. Psychol. Rev..

[93] Pearce JM, Hall G (1980). A model for Pavlovian learning: variations in the effectiveness of conditioned but not of unconditioned stimuli. Psychol. Rev..

[94] Daw ND (2018). Are we of two minds?. Nat. Neurosci..

[95] Balleine BW, Dickinson A (1998). Goal-directed instrumental action: contingency and incentive learning and their cortical substrates. Neuropharmacology.

[96] Pezzulo G, Rigoli F, Chersi F (2013). The mixed instrumental controller: using value of information to combine habitual choice and mental simulation. Front. Psychol..

[97] JASP Team. JASP (Version 0.10.0) [Computer software] (2018).

[98] van Doorn J (2019). The JASP guidelines for conducting and reporting a Bayesian analysis. PsyArXiv.

[99] Frank MJ, Moustafa AA, Haughey HM, Curran T, Hutchison KE (2007). Genetic triple dissociation reveals multiple roles for dopamine in reinforcement learning. Proc. Natl. Acad. Sci..

[100] Frank MJ, Seeberger LC, O’Reilly RC (2004). By carrot or by stick: cognitive reinforcement learning in Parkinsonism. Science.

[101] Haines N, Vassileva J, Ahn W-Y (2018). The Outcome-Representation Learning model: a novel reinforcement learning model of the Iowa Gambling Task. Cogn. Sci..

[102] Palminteri S (2009). Pharmacological modulation of subliminal learning in Parkinson’s and Tourette’s syndromes. Proc. Natl. Acad. Sci. U. S. A..

[103] Ahn W-Y, Haines N, Zhang L (2017). Revealing neurocomputational mechanisms of reinforcement learning and decision-making with the hBayesDM package. Comput. Psychiatry.

[104] Kruschke JK (2015). Doing Bayesian Data Analysis: A Tutorial with R, JAGS, and Stan.

[105] Lee MD (2011). How cognitive modeling can benefit from hierarchical Bayesian models. J. Math. Psychol..

[106] Lee MD, Wagenmakers E-J (2011). Bayesian Cognitive Modeling: A Practical Course.

[107] Rouder JN, Lu J (2005). An introduction to Bayesian hierarchical models with an application in the theory of signal detection. Psychon. Bull. Rev..

[108] Shiffrin R, Lee MD, Kim W, Wagenmakers E-J (2008). A survey of model evaluation approaches with a tutorial on hierarchical Bayesian methods. Cogn. Sci..

[109] Stan Development Team. RStan: the R interface to Stan (2018).

[110] Betancourt MJ, Girolami M, Upadhyay SK, Umesh S, Dey DK, Loganathan A (2013). Hamiltonian Monte Carlo for hierarchical models. Current Trends in Bayesian Methodology with Applications.

[111] Gelman A, Rubin DB (1992). Inference from iterative simulation using multiple sequences. Stat. Sci..

[112] Gronau QF, Wagenmakers E-J (2019). Limitations of Bayesian leave-one-out cross-validation for model selection. Comput. Brain Behav..

[113] Akaike H, Petrov BN, Csaki F (1973). Information theory as an extension of the maximum likelihood principle. 2nd International Symposium on Information Theory.

[114] Schwarz G (1978). Estimating the dimension of a model. Ann. Stat..

[115] Vehtari A, Gelman A, Gabry J (2017). Practical Bayesian model evaluation using leave-one-out cross-validation and WAIC. Stat. Comput..

[116] Nicenboim B, Vasishth S (2017). Models of retrieval in sentence comprehension: appendix. Zenodo.

