# Supplementary material for: Parallel model-based and model-free reinforcement learning for card sorting performance
Source: Sci Rep. 2020 Sep 22;10:15464. doi: 10.1038/s41598-020-72407-7 (PMC7508815; doi:10.1038/s41598-020-72407-7)
Supplement: Supplementary file 1 — Supplementary Information. [file 41598_2020_72407_MOESM1_ESM.docx]

Parallel Model-Based and Model-Free Reinforcement Learning for Card Sorting Performance

– Supplementary Materials –

Alexander Steinke^1,^*, Florian Lange^2^, and Bruno Kopp^1^

^1^ Department of Neurology, Hannover Medical School; Carl-Neuberg-Straße 1, 30625 Hannover, Germany
^2^ Behavioral Engineering Research Group, KU Leuven, Naamsestraat 69, 3000 Leuven, Belgium

* Correspondence concerning this article should be addressed to Alexander Steinke; E-mail: Steinke.Alexander@MH-Hannover

**Parameter Correlations**

Tables S1-S4 show correlations among model parameters estimated from observed data (*N* = 375). There was evidence for various correlations among estimated model parameters in all computational models under consideration. In order to examine whether these correlations were introduced by the parameter estimation process^1^, we also computed correlations among recovered model parameters estimated from simulated data (*N* = 50). Therefore, we simulated *N* = 50 cWCST runs by means of any computational model of interest using sets of randomly sampled model parameter values each. To ensure that sampled parameter values were in realistic ranges^1^, we restricted parameters to the minimum and maximum of individual parameter estimates from observed data (see Methods, Parameter estimation for details). As parameter ranges for temperature parameters were small, we extended parameter ranges for these parameters by .05.

Tables S5-S8 show correlations among model parameters estimated from simulated data (*N* = 50). For the wP-RL model, there was evidence for a correlation of the temperature parameter with the model-free learning rate after positive feedback and there was evidence for a correlation of the temperature parameter with the weighting parameter (see Table S5). For the P-RL model, there was evidence for a correlation of the temperature parameter with the model-based learning rate after negative feedback. However, these correlations are unlikely to account for the substantial correlations in model parameters estimated from observed data. Thus, these results suggest that the parameter estimation process did not introduce significant correlations between model parameters for the wP-RL and the P-RL model. In contrast, with regard to the MB-RL model, there was evidence for negative correlations of the temperature parameter with learning rates, which were also apparent in estimated parameters from observed data. Similarly, with regard to the AU model, there was evidence for a correlation of the *p*^+^ and the *d* parameter, which was also apparent in estimated parameters from observed data. Thus, parameter estimation of the MB-RL model and the AU model could have introduced correlations among model parameters. These correlations should be considered when interpreting parameter estimates of the MB-RL and the AU model. Please note that these results remain to be confirmed in a larger simulated sample^2^.

Table S1

*Correlations of wP-RL model parameters estimated from observed data (N = 375).*

|  | $\alpha_{MB}^{+}$ | $\alpha_{MB}^{-}$ | $\gamma_{MB}$ | $\alpha_{MF}^{+}$ | $\alpha_{MF}^{-}$ | $\gamma_{MF}$ | $\tau$ | *w* |
| --- | --- | --- | --- | --- | --- | --- | --- | --- |
| $\alpha_{MB}^{+}$ | - |  |  |  |  |  |  |  |
| $\alpha_{MB}^{-}$ | .361*** | - |  |  |  |  |  |  |
| $\gamma_{MB}$ | .054 | .517*** | - |  |  |  |  |  |
| $\alpha_{MF}^{+}$ | -.399*** | -.058 | .002 | - |  |  |  |  |
| $\alpha_{MF}^{-}$ | -.208** | -.239*** | -.125 | .196* | - |  |  |  |
| $\gamma_{MF}$ | .235*** | -.152 | -.100 | -.635*** | .147 | - |  |  |
| $\tau$ | -.199** | .030 | .034 | .484*** | -.061 | -.528*** | - |  |
| *w* | .248*** | .449*** | .288*** | -.284*** | -.128 | .204* | -.480*** | - |

*Note*. Pearson correlation coefficients flagged by classes of Bayes factors (BF); * 10 < BF < 100; **100 < BF < 1000; *** 1,000 < BF.

Table S2

*Correlations of P-RL model parameters estimated from observed data (N = 375).*

|  | $\alpha_{MB}^{+}$ | $\alpha_{MB}^{-}$ | $\gamma_{MB}$ | $\alpha_{MF}^{+}$ | $\alpha_{MF}^{-}$ | $\gamma_{MF}$ | $\tau$ |
| --- | --- | --- | --- | --- | --- | --- | --- |
| $\alpha_{MB}^{+}$ | - |  |  |  |  |  |  |
| $\alpha_{MB}^{-}$ | .520*** | - |  |  |  |  |  |
| $\gamma_{MB}$ | .184* | .571*** | - |  |  |  |  |
| $\alpha_{MF}^{+}$ | -.270*** | -.108 | -.041 | - |  |  |  |
| $\alpha_{MF}^{-}$ | -.190* | -.225*** | -.136 | .253*** | - |  |  |
| $\gamma_{MF}$ | .082 | -.140 | -.123 | -.464*** | .173* | - |  |
| $\tau$ | -.608*** | -.560*** | -.274*** | -.002 | -.042 | -.015 | - |

*Note*. Pearson correlation coefficients flagged by classes of Bayes factors (BF); * 10 < BF < 100; **100 < BF < 1000; *** 1,000 < BF.

Table S3

*Correlations of MB-RL model parameters estimated from observed data (N = 375).*

|  | $\alpha_{MB}^{+}$ | $\alpha_{MB}^{-}$ | $\gamma_{MB}$ | $\tau$ |
| --- | --- | --- | --- | --- |
| $\alpha_{MB}^{+}$ | - |  |  |  |
| $\alpha_{MB}^{-}$ | .519*** | - |  |  |
| $\gamma_{MB}$ | .184* | .571*** | - |  |
| $\tau$ | -.705*** | -.643*** | -.319*** | - |

*Note*. Pearson correlation coefficients flagged by classes of Bayes factors (BF); * 10 < BF < 100; **100 < BF < 1000; *** 1,000 < BF.

Table S4

*Correlations of AU model parameters estimated from observed data (N = 375).*

|  | *p^+^* | *p^-^* | *d* | *f* |
| --- | --- | --- | --- | --- |
| *p^+^* | - |  |  |  |
| *p^-^* | .175* | - |  |  |
| *d* | .276*** | .624*** | - |  |
| *f* | -.149 | .203** | .194* | - |

*Note*. Pearson correlation coefficients flagged by classes of Bayes factors (BF); * 10 < BF < 100; **100 < BF < 1000; *** 1,000 < BF.

Table S5

*Correlations of wP-RL model parameters recovered from simulated data (N = 50).*

|  | $\alpha_{MB}^{+}$ | $\alpha_{MB}^{-}$ | $\gamma_{MB}$ | $\alpha_{MF}^{+}$ | $\alpha_{MF}^{-}$ | $\gamma_{MF}$ | $\tau$ | *w* |
| --- | --- | --- | --- | --- | --- | --- | --- | --- |
| $\alpha_{MB}^{+}$ | - |  |  |  |  |  |  |  |
| $\alpha_{MB}^{-}$ | -.225 | - |  |  |  |  |  |  |
| $\gamma_{MB}$ | .061 | -.082 | - |  |  |  |  |  |
| $\alpha_{MF}^{+}$ | -.278 | -.043 | -.017 | - |  |  |  |  |
| $\alpha_{MF}^{-}$ | -.075 | -.154 | .024 | .020 | - |  |  |  |
| $\gamma_{MF}$ | .071 | -.062 | -.215 | .018 | -.236 | - |  |  |
| $\tau$ | -.001 | .034 | -.007 | -.652*** | -.369 | .044 | - |  |
| *w* | .368 | .078 | -.009 | -.281 | -.129 | .032 | -.403* | - |

*Note*. Pearson correlation coefficients flagged by classes of Bayes factors (BF); * 10 < BF < 100; **100 < BF < 1000; *** 1,000 < BF.

Table S6

*Correlations of P-RL model parameters recovered from simulated data (N = 50).*

|  | $\alpha_{MB}^{+}$ | $\alpha_{MB}^{-}$ | $\gamma_{MB}$ | $\alpha_{MF}^{+}$ | $\alpha_{MF}^{-}$ | $\gamma_{MF}$ | $\tau$ |
| --- | --- | --- | --- | --- | --- | --- | --- |
| $\alpha_{MB}^{+}$ | - |  |  |  |  |  |  |
| $\alpha_{MB}^{-}$ | .014 | - |  |  |  |  |  |
| $\gamma_{MB}$ | -.001 | .018 | - |  |  |  |  |
| $\alpha_{MF}^{+}$ | -.383 | -.037 | -.116 | - |  |  |  |
| $\alpha_{MF}^{-}$ | -.090 | -.183 | -.028 | -.097 | - |  |  |
| $\gamma_{MF}$ | -.294 | .052 | .049 | .059 | .219 | - |  |
| $\tau$ | -.264 | -.428* | .060 | -.275 | -.315 | -.058 | - |

*Note*. Pearson correlation coefficients flagged by classes of Bayes factors (BF); * 10 < BF < 100; **100 < BF < 1000; *** 1,000 < BF.

Table S7

*Correlations of MB-RL model parameters recovered from simulated data (N = 50).*

|  | $\alpha_{MB}^{+}$ | $\alpha_{MB}^{-}$ | $\gamma_{MB}$ | $\tau$ |
| --- | --- | --- | --- | --- |
| $\alpha_{MB}^{+}$ | - |  |  |  |
| $\alpha_{MB}^{-}$ | -.026 | - |  |  |
| $\gamma_{MB}$ | .026 | -.135 | - |  |
| $\tau$ | -.587*** | -.570*** | -.216 | - |

*Note*. Pearson correlation coefficients flagged by classes of Bayes factors (BF); * 10 < BF < 100; **100 < BF < 1000; *** 1,000 < BF.

Table S8

*Correlations of AU model parameters recovered from simulated data (N = 50).*

|  | *p^+^* | *p^-^* | *d* | *f* |
| --- | --- | --- | --- | --- |
| *p^+^* | - |  |  |  |
| *p^-^* | .048 | - |  |  |
| *d* | .511** | -051 | - |  |
| *f* | .212 | -.058 | -.283 | - |

*Note*. Pearson correlation coefficients flagged by classes of Bayes factors (BF); * 10 < BF < 100; **100 < BF < 1000; *** 1,000 < BF.

**Parameter Recovery**

An important prerequisite for computational models is that their parameters can be reliably recovered from observed data^1^. In order to assess parameter recovery of the studied computational models, we simulated cWCST runs as described above (Section Parameter Correlations). Please note that temperature parameters were sampled from small intervals due to restrictions of parameter ranges taken from observed data. Thus, results of parameter recovery for temperature parameters are limited by these small parameter ranges.

Figures S1-S4 show scatterplots of simulated and recovered model parameters. Parameter recovery appeared to be successful for the P-RL and the MB-RL model. In contrast, for the wP-RL model, parameter recovery should be considered insufficient for most model parameters. For the AU model, parameter recovery should be considered insufficient for the *p^+^* and the *f* parameter.

For all simulations, we restricted ranges of parameter values to those of participants’ parameter estimates. It remains possible that true parameter values are in wider ranges, which are reduced by the parameter estimation procedure. A more detailed simulation study is necessary to address this possibility. Moreover, future research should address potential effects of specific combinations of parameter values on results of parameter recovery.


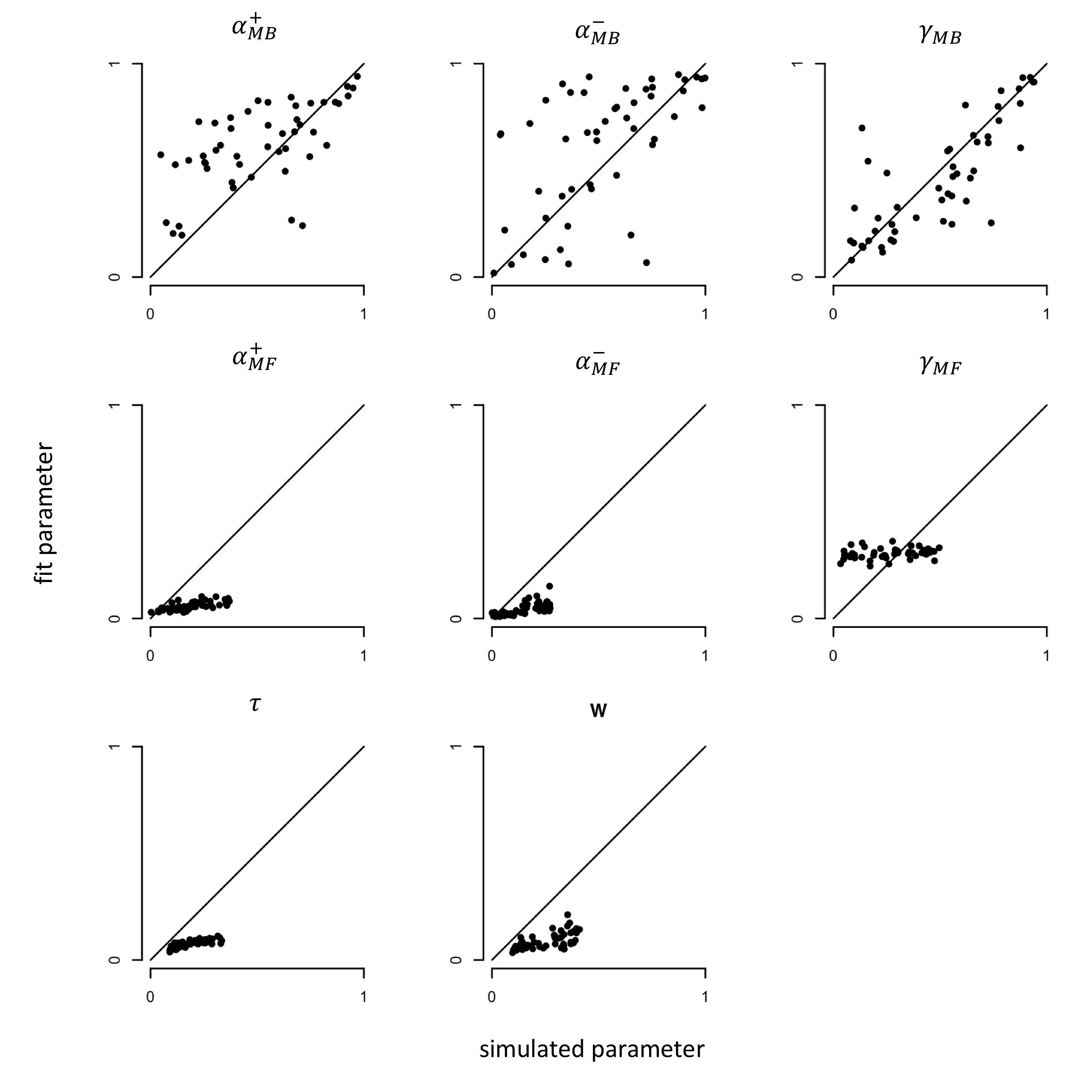


*Figure S1*. Parameter recovery of the wP-RL model.


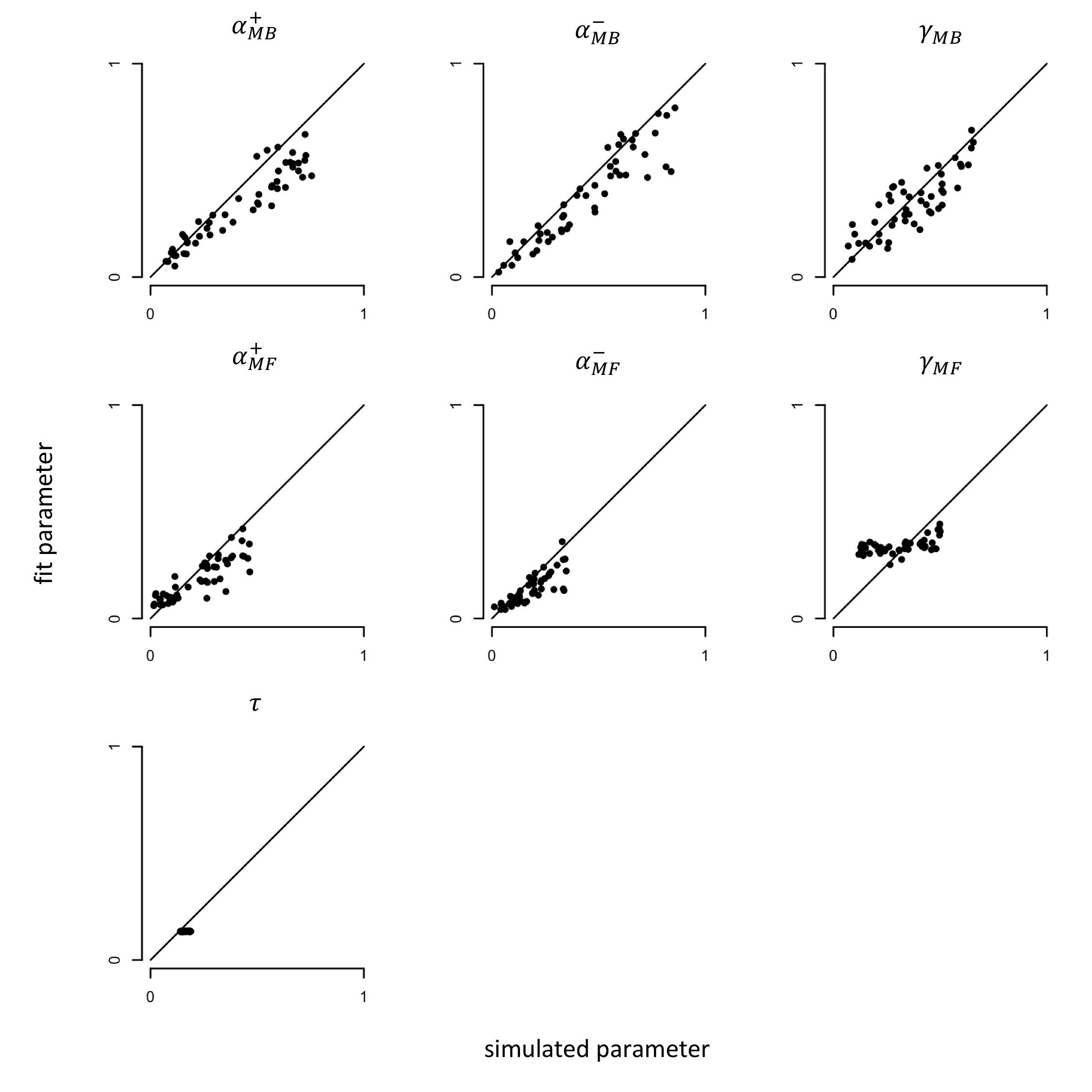


*Figure S2*. Parameter recovery of the P-RL model.


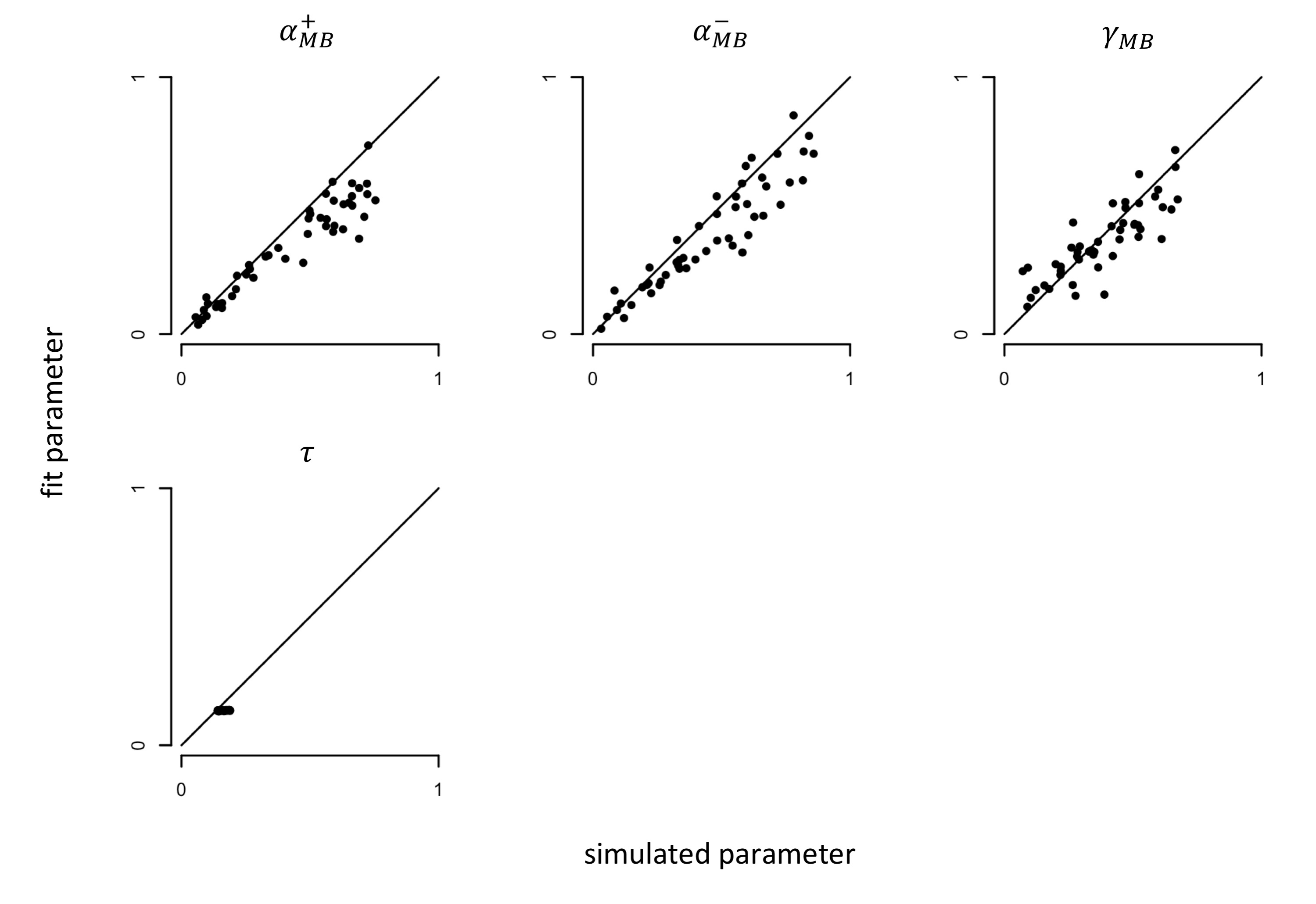


*Figure S3*. Parameter recovery of the MB-RL model.


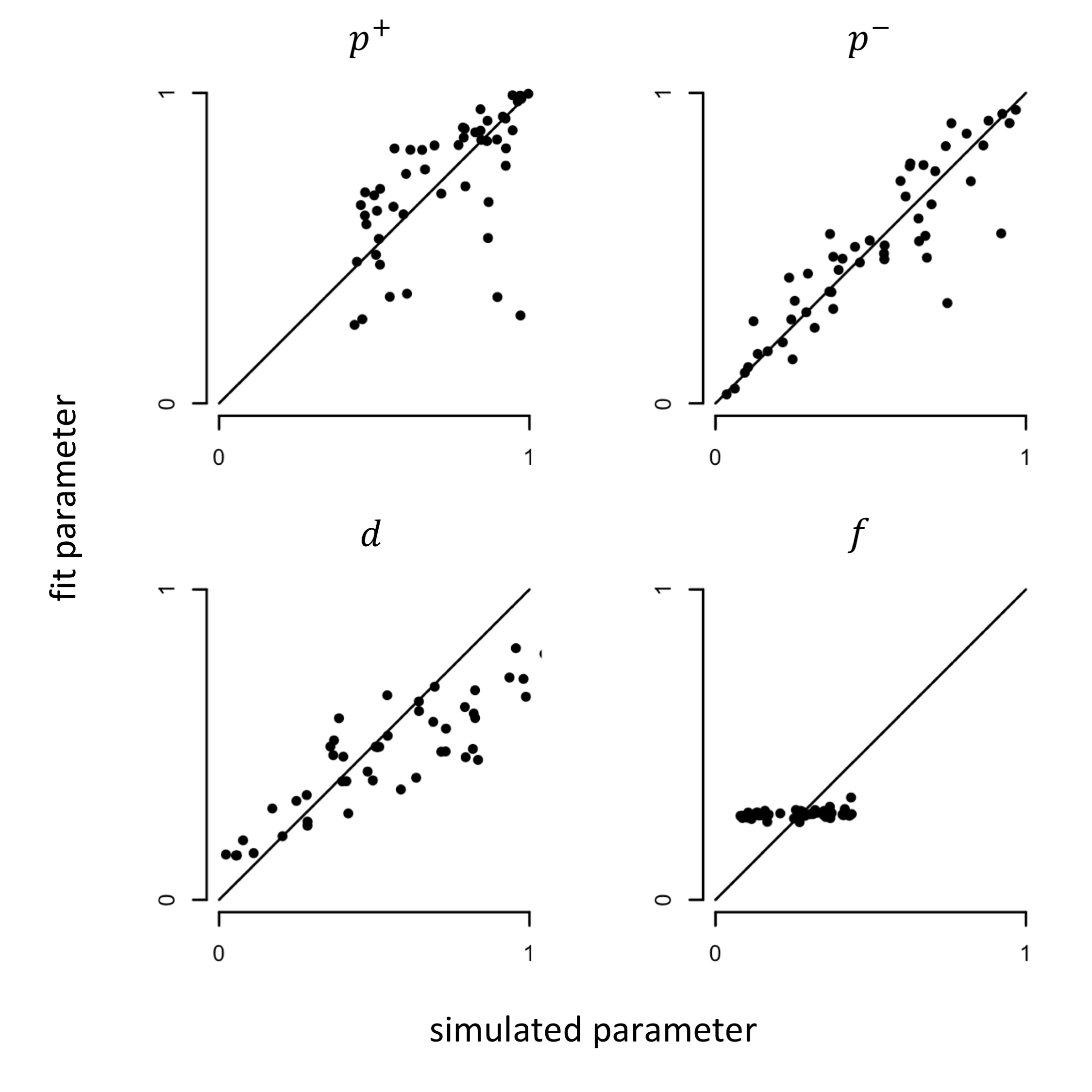


*Figure S4* Parameter recovery of the AU model.

**Model Recovery**

In order to check whether model comparisons by predictive accuracies give sensible results for simulated data^1^, we simulated cWCST behavior by any computational model as described above. Next, we computed predictive accuracies by means of any computational model under consideration for these simulated datasets. Model comparisons by predictive accuracies can be considered valid if among all competing computational models, the data-simulating model is identified as the best-fitting computational model.

Table S9

*Confusion Matrix*

| Fitted Model | Simulated Model | | | | | | | |
| --- | --- | --- | --- | --- | --- | --- | --- | --- |
|  | AU | | MB-RL | | P-RL | | wP-RL | |
| AU | 0 | (0) | -454 | (45) | -1492 | (163) | -850 | (100) |
| MB-RL | -159 | (23) | -1 | (3) | -1005 | (142) | -604 | (96) |
| P-RL | -161 | (22) | 0 | (0) | 0 | 0 | -16 | (15) |
| wP-RL | -156 | (22) | -5 | (4) | 0 | (9) | 0 | (0) |

*Note*. Depicted are estimated log predictive density (elpd) as a measure of a computational models predictive accuracy; standard error in parentheses; AU = attentional-updating model; MB-RL = only model-based reinforcement-learning model; P-RL = parallel reinforcement-learning model; wP-RL = weighted parallel reinforcement-learning model.

Results of model recovery are shown in Table S9. For any simulation, the data-simulating model performed best or was among the best performing computational models. As reinforcement-learning models were nested, multiple reinforcement-learning models could have presented similar predictive accuracies. That is, the P-RL model was nested within the wP-RL model. Furthermore, the MB-RL model was nested within the P-RL and the wP-RL model. Thus, if any of the reinforcement-learning models provided the best predictive accuracy for a given dataset, all conceptually higher reinforcement-learning models provided similarly good predictive accuracies (the slight decrease in predictive accuracies of conceptually higher reinforcement-learning models might be explained by redundant model parameters, which fit idiosyncratic noise in the data). Importantly, conceptually higher reinforcement-learning models did not perform significantly better than the data-simulating models. These results suggest that model comparisons give sensible results for simulated data. However, as mentioned above, these results remain to be confirmed in a larger simulated sample.

**Parameter Estimates of the P-RL Model**

Table S10

*Summary statistics of group-level parameter estimates of the P-RL model.*

|  |  |  |  | 95% HDI | |
| --- | --- | --- | --- | --- | --- |
| Parameter | Description | mean | *SD* | lower | upper |
| $\alpha_{MB}^{+}$ | Model-based learning rate after positive feedback | 0.56 | 0.02 | 0.52 | 0.61 |
| $\alpha_{MB}^{-}$ | Model-based learning rate after negative feedback | 0.34 | 0.02 | 0.30 | 0.37 |
| $\gamma_{MB}$ | Model-based inertia | 0.26 | 0.01 | 0.24 | 0.29 |
| $\alpha_{MF}^{+}$ | Model-free learning rate after positive feedback | < 0.01 | < 0.01 | < 0.01 | < 0.01 |
| $\alpha_{MF}^{-}$ | Model-free learning rate after negative feedback | 0.02 | < 0.01 | 0.01 | 0.03 |
| $\gamma_{MF}$ | Model-free inertia | 0.33 | 0.07 | 0.19 | 0.46 |
| $\tau$ | Inverse temperature | 0.16 | < 0.01 | 0.16 | 0.17 |

*Note*. Posterior distributions of Probit-transformed group-level location parameters from hierarchical Bayesian analysis are reported. *SD* = standard deviation; 95% HDI = 95% highest density interval.

**References**

1. Wilson, R. C. & Collins, A. G. Ten simple rules for the computational modeling of behavioral data. *Elife* **8**, (2019).

2. Schönbrodt, F. D. & Perugini, M. At what sample size do correlations stabilize? *J. Res. Pers.* **47**, 609–612 (2013).
